# Identification of Two Subgroups of Type I IFNs in Perciforme Fish Large Yellow Croaker *Larimichthys crocea* Provides Novel Insights into Function and Regulation of Fish Type I IFNs

**DOI:** 10.3389/fimmu.2016.00343

**Published:** 2016-09-07

**Authors:** Yang Ding, Jingqun Ao, Xiaohong Huang, Xinhua Chen

**Affiliations:** ^1^Key Laboratory of Marine Biogenetic Resources, Third Institute of Oceanography, State Oceanic Administration, Xiamen, China; ^2^College of Ocean and Earth Sciences, Xiamen University, Xiamen, China; ^3^Key Laboratory of Tropical Marine Bio-resources and Ecology, South China Sea Institute of Oceanology, Chinese Academy of Sciences, Guangzhou, China; ^4^Laboratory for Marine Biology and Biotechnology, Qingdao National Laboratory for Marine Science and Technology, Qingdao, China

**Keywords:** type I IFNs, antiviral immunity, IRF3 and IRF7 interaction, positive feedback regulation, promoter, large yellow croaker *Larimichthys crocea*

## Abstract

Like mammals, fish possess an interferon regulatory factor (IRF) 3/IRF7-dependent type I IFN responses, but the exact mechanism by which IRF3/IRF7 regulate the type I IFNs remains largely unknown. In this study, we identified two type I IFNs in the Perciforme fish large yellow croaker *Larimichthys crocea*, one of which belongs to the fish IFNd subgroup and the other is assigned to a novel subgroup of group I IFNs in fish, tentatively termed IFNh. The two IFN genes are constitutively expressed in all examined tissues, but with varied expression levels. Both IFN genes can be rapidly induced in head kidney and spleen tissues by polyinosinic–polycytidylic acid. The recombinant IFNh was shown to be more potent to trigger a rapid induction of the antiviral genes MxA and protein kinase R than the IFNd, suggesting that they may play distinct roles in regulating early antiviral immunity. Strikingly, IFNd, but not IFNh, could induce the gene expression of itself and IFNh through a positive feedback loop mediated by the IFNd-dependent activation of IRF3 and IRF7. Furthermore, our data demonstrate that the induction of IFNd can be enhanced by the dimeric formation of IRF3 and IRF7, while the IFNh expression mainly involves IRF3. Taken together, our findings demonstrate that the IFN responses are diverse in fish and are likely to be regulated by distinct mechanisms.

## Introduction

Teleost fish employ multiple type I IFNs to coordinate antiviral immune responses (1–3). They are classified into two major groups based on the presence of conserved cysteine residues in the mature peptide: group I IFNs containing two cysteine residues, which can be found in all teleost fish lineages, and group II IFNs containing four cysteine residues which are limited in several species, such as trout *Oncorhynchus mykiss*, salmon *Salmo salar*, and zebrafish *Danio rerio* (2, 4, 5). Phylogenetically, the two groups can be further divided into six subgroups, IFN a, b, c, d, e, and f (2). Notably, to date, the Perciformes, such as sea bass *Dicentrarchus labrax* (6), rock bream *Oplegnathus fasciatus* (7), sevenband grouper *Epinephelus septemfasciatus* (8), and orange-spotted grouper *Epinephelus coioides* (9) have been shown to possess a single subgroup, IFNd.

The antiviral functions of fish type I IFNs have been characterized in multiple fish species. As in mammals, fish type I IFNs are able to induce expression of a variety of antiviral genes, including myxovirus resistance (Mx), protein kinase R (PKR), virus inhibitory protein, endoplasmic reticulum-associated, IFN-inducible (Viperin), and IFN-stimulated gene (ISG) 15, thus leading to an enhanced antiviral state (4, 10–12). Accumulating data suggest that fish group I and II type I IFNs may have distinct antiviral roles in different cells or at different stages of infection (3). For example, zebrafish IFNphi1 (IFNa, group I) induces a slow and powerful expression of antiviral genes, whereas zebrafish IFNphi2/3 (IFNcs, group II) trigger a rapid and transient induction of antiviral genes (5). In zebrafish larvae, IFNphi4 (IFNd, group I) exhibits poor antiviral activity (10). Consistent with these reports, salmon IFNa, but not IFNd, exerts significant antiviral effects (11). In contrast to these reports, in Perciforme species, such as rock bream, sevenband grouper, and orange-spotted grouper, IFNds (group I) are the main IFNs to mount antiviral defense to viral infection (7–9).

In general, teleost group I type I IFNs appear to be ubiquitously expressed in most cell types and tissues and are upregulated upon viral infection or viral RNA analog treatment, whereas group II type I IFNs are constitutively expressed at a very low level and induced in specific leukocyte populations, with the exception of IFNf, which can be induced in fibroblasts (2, 3, 13). Recent studies demonstrate that the six IFN subgroups in trout were differentially modulated in three trout cell types, RTG-2, RTS-11, and primary head kidney leukocytes, following stimulation with polyinosinic–polycytidylic acid [poly(I:C)]. Moreover, viral haemorrhagic septicemia virus infection of brown trout *Salmo trutta* also gave rise to differential expression kinetics in the kidney and spleen (2). Similar findings have been reported for zebrafish, salmon, and turbot *O. fasciatus* type I IFNs (10, 11, 14). These differential expression patterns of IFNs between or within group I and group II suggest that regulation of type I IFN expression in fish is very complex.

The expression of type I IFNs is controlled by two key transcription factors, the interferon regulatory factor (IRF) 3 and 7 (15). In mammals, viruses are recognized by pattern recognition receptors, including Toll-like receptors (TLRs) and retinoic acid–inducible gene I-like receptors (RLRs), which trigger distinct signaling cascades to activate IRF3 and/or IRF7, inducing expression of early phase IFNs mainly, including IFNβ. IFNβ, then, induces expression of a variety of ISGs to establish the host antiviral state through the Jak–Stat pathway and the IRF7-dependent production of the late-phase IFNs, including most of the IFNαs (16–18). Similar to mammals, fish IFN responses are also controlled by IRF3/7 and appear to be very complex. Accumulating data suggest that fish group I and group II IFN responses are governed by distinct IRFs. Group I IFN genes, including zebrafish IFNphi1, carp *Carassius auratus* IFN, and salmon IFNa1, as well as Japanese flounder *Paralichthys olivaceus* IFN (IFNd), seem to be primarily regulated by IRF3, while expression of zebrafish IFNphi3 (group II) mainly involves IRF7 (19–21). Recent studies show that zebrafish IFNphi1 and salmon IFNa1 are also activated by IRF1 and IRF7, respectively (19, 22). Furthermore, fish IFNas can significantly induce the expression of themselves and other IFN genes (5, 23), suggesting that a positive feedback regulation may exist. This observation differs from that of mammals, in which type I IFNs cannot directly induce their own expression (24, 25). Further studies demonstrate that the carp IFN facilitates phosphorylation of IRF3 that is required for activation of gene transcription, thus amplifying IFN response (20). However, the roles of the IRF3 and IRF7 in the positive feedback regulation remain largely unknown.

In this study, we report the identification of two type I IFNs from large yellow croaker (lyc) *Larimichthys crocea*. Based on the sequence and phylogenetic analyses, one IFN belonged to the IFNd subgroup while the other was assigned to a novel subgroup of group I IFNs, tentatively termed IFNh. Lyc IFNd and IFNh exhibited apparent differences in expression patterns and the ability to induce antiviral genes. IFNd, but not IFNh, was able to upregulate expression of itself and IFNh, as well as the activation of phosphorylation of IRF3 and IRF7. Furthermore, expression of the IFNd gene requires both IRF3 and IRF7, while the IFNh expression primarily involves IRF3. Collectively, the lyc IFNd may function as a key mediator for amplification of the IFN responses through IRF3 and IRF7. These findings provide new insights into the function and regulation of type I IFNs in fish.

## Materials and Methods

### Ethics Statement

The studies were carried out in strict accordance with the Regulations of the Administration of Affairs Concerning Experimental Animals, under protocol license number: SYXK(MIN)2007-0004, approved by the Institutional Animal Care and Use Committee of Fujian Province. All of the surgery was performed under Tricaine-S anesthesia, and all efforts were made to minimize suffering.

### Fish

Large yellow croaker *L. crocea* (lyc, weight: 103 ± 21.9 g; length: 21 ± 1.3 cm) were purchased from a mariculture farm in Lianjiang county, Fuzhou, China. Fish were maintained with a flow-through seawater supply at 25°C. After acclimating for 7 days, healthy fish were used for the challenge experiments.

### Cells Lines and Virus

The lyc head kidney (LYCK) cells were isolated from the head kidney of lyc. The continuous LYCK cell lines were preserved in our laboratory and maintained at 28°C in L-15 medium (Life Technologies, Carlsbad, CA, USA), supplemented with 10% Fetal Bovine Serum (FBS, Life Technologies) according to the previous study (26). The epithelioma papulosum cyprini (EPC) cells (China Center for Type Culture Collection, Wuhan, China) were derived from fathead minnow *Pimephales promelas* and cultured at 25°C in L-15 medium supplemented with 10% FBS (27). Human embryonic kidney 293T cells (HEK293T, China Center for Type Culture Collection) were grown in DMEM (Life Technologies) containing 10% FBS, 100 U/ml penicillin, and 100 μg/ml streptomycin (Life Technologies) at 37°C in a 5% CO_2_ atmosphere. Grouper spleen (GS) cells were originated from the spleen of orange-spotted grouper *E. coioides* and maintained in L-15 medium supplemented with 10% FBS at 25°C. Singapore grouper iridovirus (SGIV) was propagated in GS cells as previously described (28), and the virus stock was stored at −80°C until use. GS cells and SGIV are generous gifts from Professor Qiwei Qin in South China Sea Institute of Oceanology, Chinese Academy of Sciences.

### Gene Cloning and Bioinformatics

The partial sequences of lyc IFNd and IFNh were obtained from the transcriptome library of lyc spleen tissues (29). 5′ and 3′ RACE PCR were performed to obtain the full-length cDNAs of IFNd and IFNh, as described previously (30). The cDNA for 5′ and 3′ RACE PCR was derived from the LYCK sampled at 6 h after stimulation with poly(I:C). The integrity of the cDNA sequences was confirmed by PCR with the primers covering the full-length coding sequence (Table S1 in Supplementary Material). The genomic sequence and 5′-flanking regulatory sequence of IFNd and IFNh were obtained from the lyc genome data (31) and amplified from genomic DNA of the lyc muscle with specific primers (Table S1 in Supplementary Material).

Amino acid sequence identity and similarity were calculated using the Matrix Global Alignment Tool (Matgat, version 2.0) (32). Multiple alignments were performed with CLUSTAL W2 program, and phylogenetic trees were constructed by the Neighbor-Joining and Minimum Evolution methods using the MEGA (version 6) software package. Signal peptide predictions were made using SignalP4.1 software.^1^ The genomic organization of IFNd and IFNh genes was analyzed by alignment of the IFN cDNA sequences and their genomic DNA sequences using Spidey program.^2^ Transcription factor binding sites were predicted using the MatInspector program.^3^

The fish IFN sequences retrieved from the databases for analysis included: *C. auratus* (*Ca*, goldfish), AAR20886; *Cirrhinus molitorella* (*Cm*, mud carp), AAY56128; *Ctenopharyngodon idella* (*Ci*, grass carp), ABC87312; *Cyprinus carpio* (*Cc*, common carp), ADI81047; *D. rerio* (*Dr*, zebrafish), AAM95448 (IFNphi1), NP_001104552 (IFNphi2), NP_001104553 (IFNphi3), NP_001155212 (IFNphi4); *D. labrax* (*Dl*, sea bass), CAQ17043 (IFN1); *E. coioides* (*Ec*, orange-spotted grouper), AGL21770 (IFN1), AGJ98284 (IFN2); *Gasterosteus aculeatus* (*Ga*, stickleback), CAM31706 (IFN1), CAM31707 (IFN2), CAM31708 (IFN3); *Haplochromis burtoni* (*Hb*, Burton’s mouthbrooder), XP_005950669 (IFNal3); *Ictalurus punctatus* (*Ip*, catfish), AAV97701 (IFN), AAV97699 (IFN2); *Maylandia zebra* (*Mz*, zebra mbuna), XP_004556871 (IFNal3); *Mylopharyngodon piceus* (*Mp*, black carp), AKM15287; *O. mykiss* (*Om*, trout), CAM28541 (IFNa1), NP_001153977 (IFNa2), CCV17397 (IFNa3), CCV17398 (IFNa4), NP_001153974 (IFNb1), NP_001158515 (IFNb2), CCV17399 (IFNb3), CCV17400 (IFNb4), CCV17401 (IFNb5), CCV17402 (IFNc1), CCV17403 (IFNc2), CCV17404 (IFNc3), CCV17405 (IFNc4), CAV07949 (IFNd1), CCV17406 (IFNe1), CCV17407 (IFNe2), CCV17408 (IFNe3), CCV17409 (IFNe4), CCV17410 (IFNe5), CCV17411 (IFNe6), CCV17412 (IFNe7), CCV17413 (IFNf1), CCV17414 (IFNf2); *O. fasciatus* (*Of*, turbot), AFP94213 (IFN1), AFP94213 (IFN2); *Oreochromis niloticus* (*On*, tilapia), XP_005950669 (IFNω1), XP_005469255 (IFNω3), XP_003453450 (IFNal3); *Oryzias latipes* (*Ol*, medaka), BAU25609 (IFN1); *P. olivaceus* (*Po*, Japanese flounder), BAA02372; *Pundamilia nyererei* (*Pn*, cichlid), XP_013771349 (IFNal3); *S. salar* (*Ss*, salmon), ABD39320 (IFNa1), ABD39321 (IFNa2), ACE75687 (IFNa3), ACE75691 (IFNb1), ACE75693 (IFNb2), ACE75689 (IFNb3), ACE75692 (IFNc1), XP_014048249 (IFNc2), ACE75688 (IFNc3), DAA64377 (IFNd); *Sparus aurata* (*Sa*, gilthead seabream), CAT03221 (IFN1), CAT03222 (IFN2), CAT03223 (IFN3), CAT03224 (IFN4); *Takifugu rubripes* (*Tr*, Fugu), CAM82750 (IFN1), CAM82751 (IFN2); *Tetraodon nigroviridis* (*Tn*, spotted green pufferfish), CAD67779.

### Production of Recombinant lyc IFN Proteins

To obtain the recombinant IFN (rIFN) proteins, the coding sequences of IFNd and IFNh, with the signal peptide deleted, were inserted into the pCMV-Flag 2C vector (Stratagene, La Jolla, CA, USA) using gene-specific primer sets (Table S1 in Supplementary Material) and expressed as a fusion protein with the FLAG tag in HEK293T cells. 3 × 10^6^ HEK293T cells were plated in 9-cm tissue culture dishes (Biofil, Guangzhou, China) and transfected with 18 μg of rIFN plasmid using 36 μl of Fugene^®^ HD transfection reagent (Promega, Madison, WI, USA). At 48 h after transfection, cells were harvested for analysis of the expression of rIFN proteins. The recombinant proteins were then purified using ANTI-FLAG^®^ M2 affinity gel (Sigma-Aldrich, St. Louis, MO, USA) according to the manufacturers’ instructions. Briefly, the harvested cells were lysed with the lysis buffer [TBS (50 mM Tris–HCl, pH 7.4, 150 mM NaCl), 1 mM EDTA, and 1% Trition X-100] and incubated with ANTI-FLAG^®^ M2 affinity gel for 1 h at 4°C. Then the beads were washed with TBS, and the recombinant proteins eluted with TBS containing 3 × FLAG peptides (200 ng/μl, Sigma-Aldrich). After dialyzed against phosphate-buffered saline (PBS; 137 mM NaCl, 2.7 mM KCl, 10 mM Na_2_HPO_4_, 2 mM KH_2_PO_4_, pH7.4), the purified proteins were concentrated using an ultrafiltration centrifuge tube (Millipore, Bedford, MA, USA) and stored at −70°C after filtration with a 0.45-μM filter. The purified rIFN proteins were quantitated using Bradford protein quantitation assay by Nanodrop 1000 (Thermo Fisher Scientific, Waltham, MA, USA).

### Antiviral Activity Assays in Grouper Spleen Cells

The GS cells were seeded onto the 6-well plates (Thermo Fisher Scientific) for 18 h. The cells were pretreated with rIFNd or rIFNh at a final concentration of 50 ng/ml or PBS (as a control) for 2 h; then, the cells were infected with SGIV at a multiplicity of infection of 2. At 24 h postinfection, the cells were observed microscopically for cytopathic effect (CPE) (Leica Microsystems, Wetzlar, Germany).

The expression of two SGIV envelope protein genes, ORF049 and ORF072, was detected by real-time PCR. Briefly, infected cells were harvested at 24 and 48 h postinfection. Total RNA was extracted using the SV total RNA Isolation System (Promega) according to the manufacturer′s instructions, and reverse-transcribed into first-strand cDNA using an Oligo dT-Adaptor primer (TaKaRa, Dalian, China). Real-time PCR was performed with gene-specific primer sets (Table S1 in Supplementary Material). *E. coioides* β-actin (*Ec*β-actin) was amplified as an internal control with the *Ec*actin-F/*Ec*actin-R primers (Table S1 in Supplementary Material). Real-time PCR was performed on the Mastercycler ep gradient realplex4 system (Eppendorf, Germany) using SYBR^®^ Premix ExTaq™ (TaKaRa). Cycling conditions were 3 min at 94°C, then 40 cycles at 94°C for 5 s, 60°C for 10 s, and 72°C for 10 s. The fluorescence output for each cycle was analyzed upon the completion of the entire run. The expression levels of SGIV genes, ORF049 and ORF072, were normalized by *Ec*β-actin using the 2^–ΔΔCT^ method (33). Each experiment was repeated three times.

### Expression Analysis of lyc IFN Genes

To determine the tissue expression profiles of IFN genes, tissues including brain, gills, heart, head kidney, intestine, liver, skin, spleen, and stomach were collected from five healthy lyc fish. Total RNA was isolated using the Trizol reagent (Life Technologies) and treated with RNase-free DNase I (TaKaRa). After reverse transcription, real-time PCR was carried out using gene-specific primer sets (Table S1 in Supplementary Material) and the cycling conditions were 30 s at 95°C, followed by 40 cycles at 95°C for 5 s, 58°C for 15 s, and 72°C for 20 s. The expression levels of IFN genes were normalized by β-actin using the 2^–ΔΔCT^ method as above and expressed as the ratio of the IFNd expression levels in the spleen.

To understand the modulation of IFN gene expression upon poly(I:C) challenge, one group of 25 fish was intraperitoneally injected with poly(I:C) (Sigma-Aldrich, St. Louis, MO, USA; 1 mg/ml in PBS) at a dose of 0.2 mg/100 g fish. Another group of 25 fish was injected with sterile PBS at a dose of 0.2 ml/100 g fish as a control. The head kidney and spleen were collected from five fish in each group at 4, 8, 12, 24, and 48 h postinjection, frozen immediately in liquid nitrogen, and stored at −80°C until RNA extraction. Total RNA was extracted from head kidneys and spleens collected at the described time points postinjection. Real-time PCR was then performed using the conditions described above to detect the expression levels of two IFN genes at different time points postinjection. The relative expression levels of IFN genes were normalized by the reference gene β-actin. Fold change of gene expression level was obtained by comparing the normalized gene expression level of poly(I:C)-injected fish with that of the PBS-injected fish (defined as 1) at the same time point.

### Treatment of LYCK Cells with rIFN Proteins

To determine the bioactivities of IFNd and IFNh, the LYCK cells were plated in 6-well plates with a density of 1 × 10^6^ cells/well and treated with rIFNs at a final concentration of 50 ng/ml or PBS (as a control). Three replicate wells were used for each treatment. The LYCK cells were harvested at 0, 2, 4, 8, and 20 h posttreatment, and total RNA was extracted as described above. Expression levels of lyc IFNd, IFNh, MxA, PKR, IRF3, and IRF7 genes were determined using real-time PCR as described previously. Fold change of gene expression level was calculated by comparing the normalized gene expression level in rIFN-treated cells with that in PBS-treated cells (defined as 1) at the same time point. Each experiment was repeated three times.

### Luciferase Activity Assay

For luciferase assays, the recombinant plasmids were constructed by inserting the promoter regions of two IFN genes and a series of their respective deleted fragments into the dual luciferase reporter plasmid pGL3-Basic (pGL3-IFNPs, primers in Table S1 in Supplementary Material; Promega). The EPC cells (5 × 10^4^/well) were seeded in 96-well plates (Thermo Fisher Scientific) overnight and cotransfected with 100 ng of pGL3-IFNP plasmid or pGL3-Basic plasmid (control) and 2 ng pRL-TK plasmid using the Fugene^®^ HD transfection reagent. After 48 h, the luciferase activity of total cell lysates was measured on a GloMax 20/20 luminometer (Promega) according to the Dual-Luciferase^®^ Repoter Assay System (Promega). The firefly luciferase activity was normalized to the Renilla luciferase activity (pRL-TK, Promega), and the IFNP relative luciferase activity (IFNP Rel. Luci. Act.) was expressed as the ratio of normalized luciferase activity in cells transfected with pGL3-IFNPs versus that in control cells transfected with the pGL3-Basic plasmid. To further study the effect of IRF3 and IRF7 on IFN promoter activity, the complete ORFs of lyc IRF3 and IRF7 were cloned into the pCMV-HA vector (pCMV-HA-IRFs, primers in Table S1 in Supplementary Material). The resulting plasmids [pGL3-IFNPs (50 ng), pCMV-HA-IRF (50 ng)/pCMV-HA (50 ng), and pRL-TK (1 ng)] were cotransfected into the EPC cells. The IFNP relative luciferase activity was expressed as the ratio of normalized luciferase activity in cells cotransfected with pGL3-IFNPs and pCMV-HA-IRF3/7 versus that in control cells cotransfected with pGL3-Basic and pCMV-HA plasmids. All data were obtained from three independent experiments with each performed in triplicate.

### Preparation of IRF3 and IRF7 Polyclonal Abs

To produce the polyclonal anti-IRF3 and anti-IRF7 antibodies (Abs), the DNA binding domains (DBD; IRF3^1-113aa^, *KKF34018*; IRF7^1-110aa^, *KKF30244*) were amplified using gene-specific primers (Table S1 in Supplementary Material) and inserted into the pET-32a vector. The recombinant proteins were expressed in *E. coli* BL21 (Novagen, Madison, WI, Germany) as a fusion protein and purified as described previously (30). The purified proteins were injected into the white New Zealand rabbits to raise polyclonal Abs using the standard method (34). The polyclonal Abs were pre-adsorbed using *E. coli* lysate supernatants to remove the irrelevant Abs and purified using the HiTrap™ Protein A HP system on AKTAprime™ Plus (GE Healthcare, Piscataway, NJ, USA).

### Western Blotting

To determine the specificity of the rabbit anti-IRF3 and anti-IRF7 Abs prepared above, HEK293T cells were transfected with pCMV-HA-IRF3, pCMV-HA-IRF7, and pCMV-HA (Clontech, as control) for 48 h. The lysates of the transfected cells were separated by 12% SDS-PAGE and electrophoretically transferred to a PVDF membrane (Millipore). The membrane was blocked in 5% (w/v) non-fat milk in TBST buffer (25 mM Tris–HCl, pH 7.4, 137 mM NaCl, 2.7 mM KCl, and 0.05% Tween 20) at 25°C for 1 h, incubated with primary Abs [rabbit anti-IRF3 or anti-IRF7 Abs (1:1000); or rat anti-HA Ab (1:8000, Sigma-Aldrich)] at 4°C overnight, then incubated with HRP-conjugated secondary Abs (goat anti-rabbit Ab, 1:3000, Sigma-Aldrich; or goat anti-rat Ab, 1:5000, Solarbio, Beijing, China) for 1 h at 25°C. All washing operations were performed on the SNAP i.d. system (Millipore) using three TBST buffer washes. The membrane was stained using the ECL system.

To detect the endogenous IRF3 and IRF7, the LYCK cells were cultured in a 6-cm plate (2.5 × 10^6^) and treated with poly(I:C) and rIFNd or rIFNh at a range of doses for 12 h. Total protein was incubated with or without 20 U of calf intestinal alkaline phosphatase (CIAP) at 37°C for 30 min and, then, separated by 12% SDS-PAGE and transferred to a PVDF membrane using PierceG2 Fast Blotter equipment (25V for 10 min; Pierce, Rockford, IL, USA). The primary Abs (rabbit anti-IRF3 and anti-IRF7 Abs, 1:1000) were incubated using 1% (w/v) non-fat milk in TBST buffer (0.1% Tween 20). The secondary Ab was HRP-conjugated goat anti-rabbit Ab (1:3000). Immunoreactive proteins were detected using an ECL system.

### Co-immunoprecipitations

To detect the interaction of lyc IRF3 and IRF7, complete ORF of IRF3 was cloned into the pCMV-Flag 2C vector (pCMV-Flag-IRF3, primers in Table S1 in Supplementary Material). The immunoprecipitation method for analysis of IRF3 and IRF7 was performed using ANTI-FLAG^®^ M2 affinity gel (agarose beads conjugated with murine anti-Flag monoclonal Ab) according to the manufacturers’ instructions. In brief, 3 × 10^6^ HEK293T cells were seeded in 9-cm tissue culture dishes overnight and then cotransfected with 1.8 mg of pCMV-Flag-IRF3 and pCMV-HA-IRF7 (at a ratio of 1:1) using 36 μl of Fugene^®^ HD transfection reagent. Cells transfected with pCMV-Flag-IRF3/pCMV-HA, pCMV-Flag/pCMV-HA-IRF7 or empty vectors were used as controls. At 48 h after transfection, cells were harvested and lysed with cell lysis buffer (Beyotime, Nantong, China). The IRF3-Flag immune complexes were then immune-precipitated from supernatants of cell lysates using ANTI-FLAG^®^ M2 affinity gel for 1 h at 4°C. The beads were washed with cell lysis buffer for five times and eluted by boiling beads in 5 volumes of SDS-PAGE loading buffer. Finally, the samples, including controls, were used for SDS-PAGE and Western blotting analyses using anti-Flag Ab or anti-HA Ab (Sigma-Aldrich) against the fusion protein.

### EMSA

EMSA was performed as previously described (35). Briefly, the lysates of HEK293T cells transfected with pCMV-HA-IRF3 or pCMV-HA-IRF7 were prepared for DNA–protein binding reactions. The wild-type and mutated oligonucleotides (Table S1 in Supplementary Material) for EMSA probes were biotin-labeled using an EMSA Probe Biotin Labeling Kit (Beyotime) according to the manufacturer’s instructions. DNA–protein binding reactions were carried out using an EMSA/Gel-Shift Kit (Beyotime) at 25°C according to the manufacturer’s instructions. In parallel, to determine the specificity of the DNA–protein binding reactions, competition experiments were performed with 100 × excessive unlabeled wild-type or mutated probes. After a 20 min incubation, the completed reactions were separated by non-denaturing 4% PAGE, and the gel was subjected to autoradiography using a LightShift^®^ Chemiluminescent EMSA Kit (Pierce).

## Results

### Gene Cloning and Sequence Analysis of Two lyc IFNs

**Figure 1:**
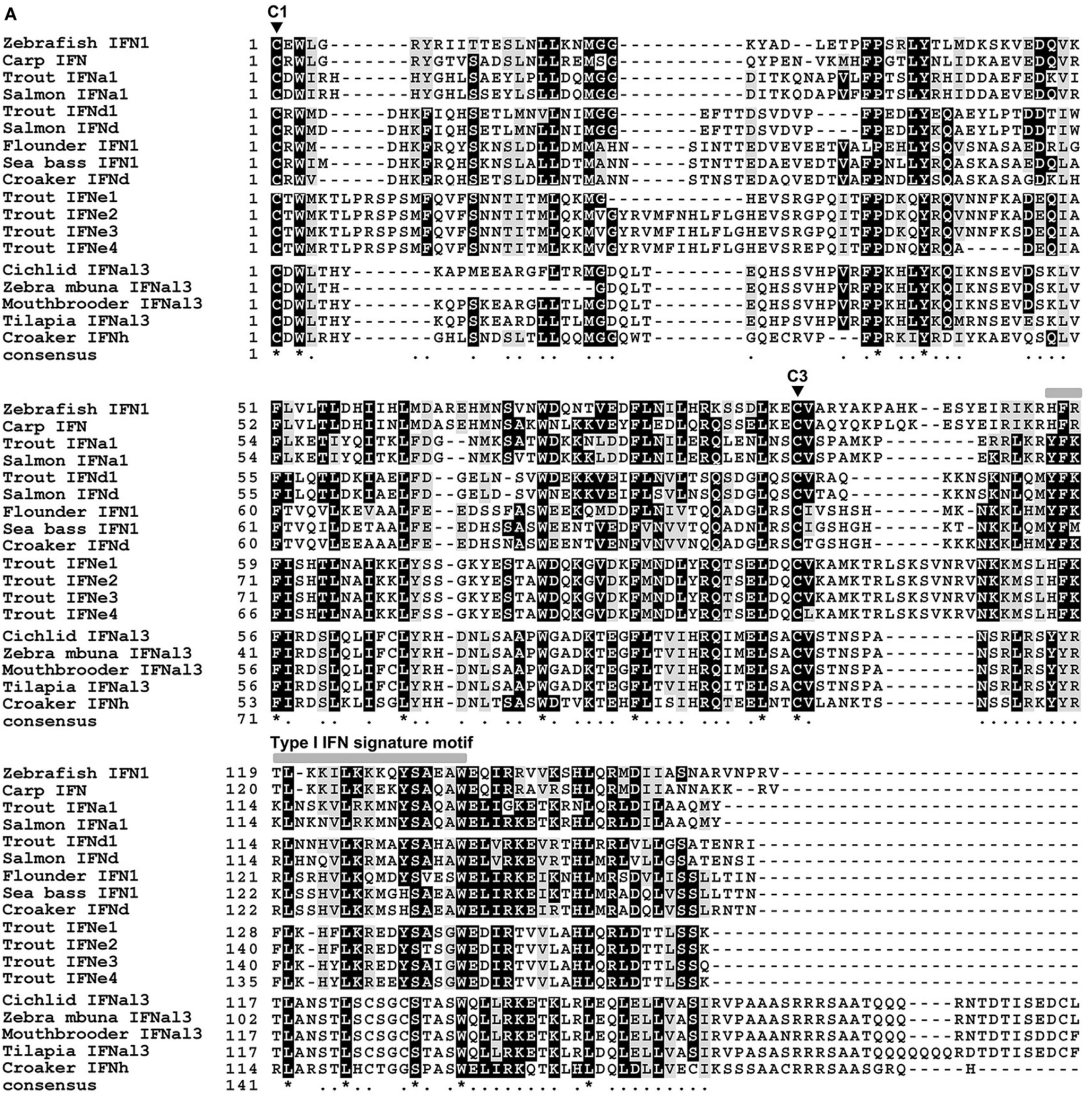

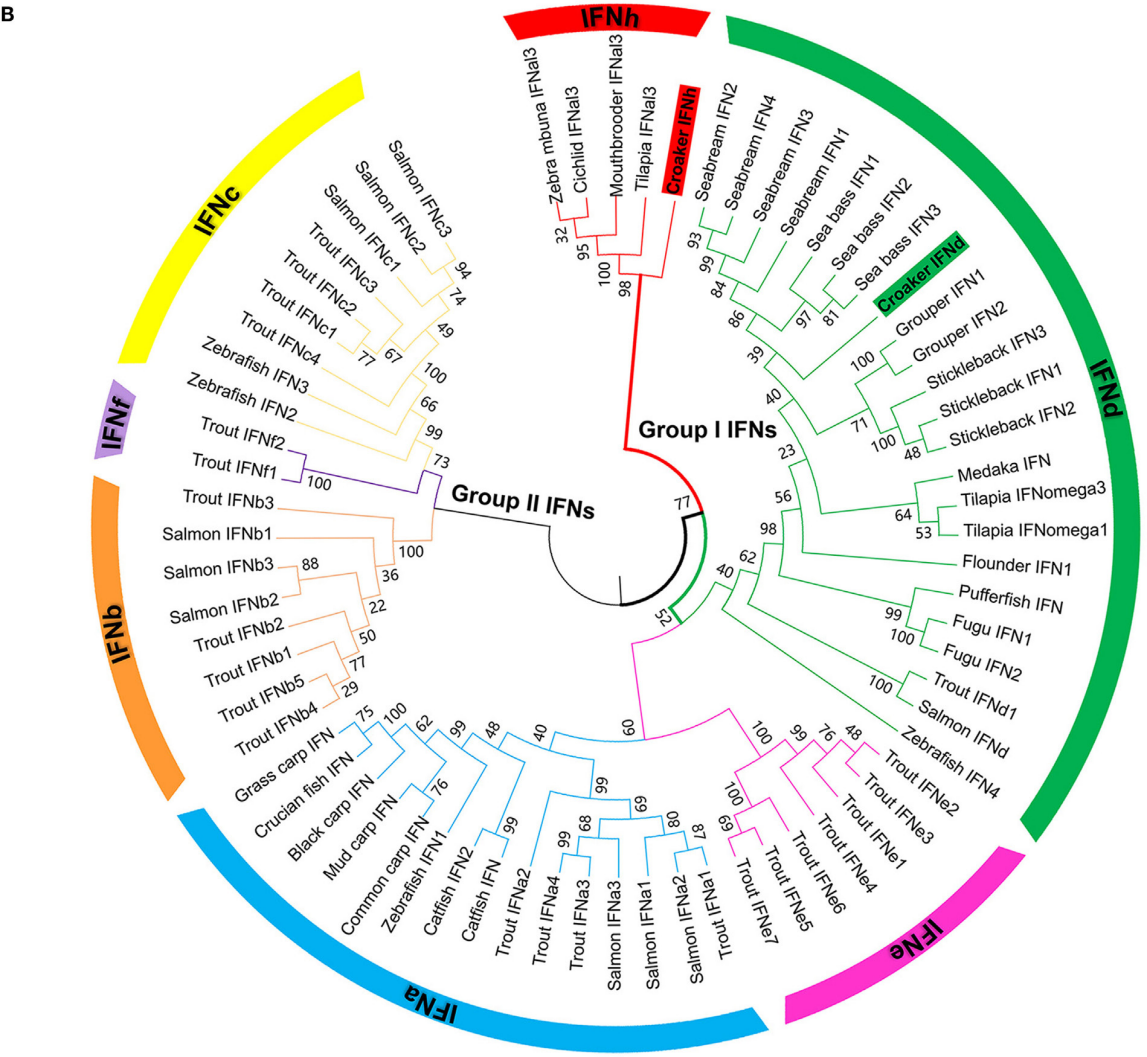
**Sequence and phylogeny analysis of fish IFNs**. **(A)** Multiple alignment of mature peptide sequences of group I type I IFNs (IFNa, d, e, and h) from large yellow croaker and other teleosts. Sequence alignments were obtained using CLUSTAL W2 program, and the conserved residues are shaded using BOXSHADE (v3.21). The two highly conserved cysteine residues of group I type I IFNs (C1 and C3) are indicated by triangles. The signature motif of type I IFNs are marked above the alignment. Identical residues are indicated by stars, while similar residues are indicated by single dots. **(B)** Phylogenetic tree of fish type I IFN family members based on the genetic distances of deduced amino acid sequences. Deduced amino acid sequences of type I IFN family members were aligned, and the tree was constructed with the Neighbor-Joining method using the MEGA (version 6) software package. The tree is bootstrapped 10,000 times, and the bootstrap values of the major branches are shown as percentages.

The lyc IFNd (*KU144879*) and IFNh (*KU144880*) genes were identified and their coding sequences determined. The complete IFNd cDNA is 934 bp in length, with an open reading frame (ORF) translating into a protein of 185 aa, where a signal peptide of 22 aa can be predicted. The deduced IFNd protein contains two cysteine residues (C1: C^23^ and C3: C^125^) conserved in the mature peptides of fish group I type I IFNs (Figure 1A, Figure S1A in Supplementary Material). The full-length cDNA of IFNh is 822 bp, containing an ORF encoding a protein of 190 aa, with a predicted signal peptide of 21 aa. Although the deduced IFNh protein contains six cysteine residues, only two are aligned with the conserved cysteine residues (C1: C^22^ and C3: C^108^) in the fish group I type I IFNs (Figure 1A, Figure S1B in Supplementary Material). Both lyc IFNs possess some typical features of type I IFNs in teleost fish, including a distinctive family signature motif ([FYH]-[FY]-X-[GNRCDS]-[LIVM]-X^2^-[FYL]-L-X^7^-[CY]-[AT]-W) at the C-terminus and a gene organization of five exons and four introns (Figures S1A,B in Supplementary Material).

The phylogenetic relationships of the lyc IFNs with other fish IFN homologs were studied. The phylogenetic tree with the Neighbor-Joining method shows that lyc IFNd falls into a major clade with the fish IFNd subgroup (Figure 1B). To our surprise, the lyc IFNh does not cluster with any known group I type I IFNs (IFNa, IFNd, and IFNe), but forms a separate clade with IFNs from zebra mbuna *M. zebra*, Burton’s mouthbrooder *H. burtoni*, tilapia *O. niloticus*, and Nyerere’s Victoria cichlid *P. nyererei* (Figure 1A, Table S2 in Supplementary Material), which is likely to represent a novel subgroup of group I IFNs. Additionally, the phylogenetic tree constructed by the minimum evolution method gives a similar tree topology (Figure S2 in Supplementary Mateiral).

Multiple sequence alignment revealed that all of the IFNh members share a well-conserved signature motif, which has some amino acid variation compared with other group I IFN members (Figure 1A). Homology comparison showed that lyc IFNd exhibits the highest sequence identity of 82.8% with sea bass IFNd, followed by 82.3–66% identity with IFNd members from other Perciforme species, whereas a low sequence identity of 17.1–31.7% with members of other subgroups (Table S2 in Supplementary Material). Lyc IFNh shares 48.4–55.2% sequence identity to its homologs in other fish species, but only 17.2–35.1% to those of other subgroups (Table S2 in Supplementary Material). These results further support the classification of IFNh as a novel subgroup, which is distinct from the six subgroups of type I IFNs already known.

### Expression Analysis of lyc IFN Genes

**Figure 2:**
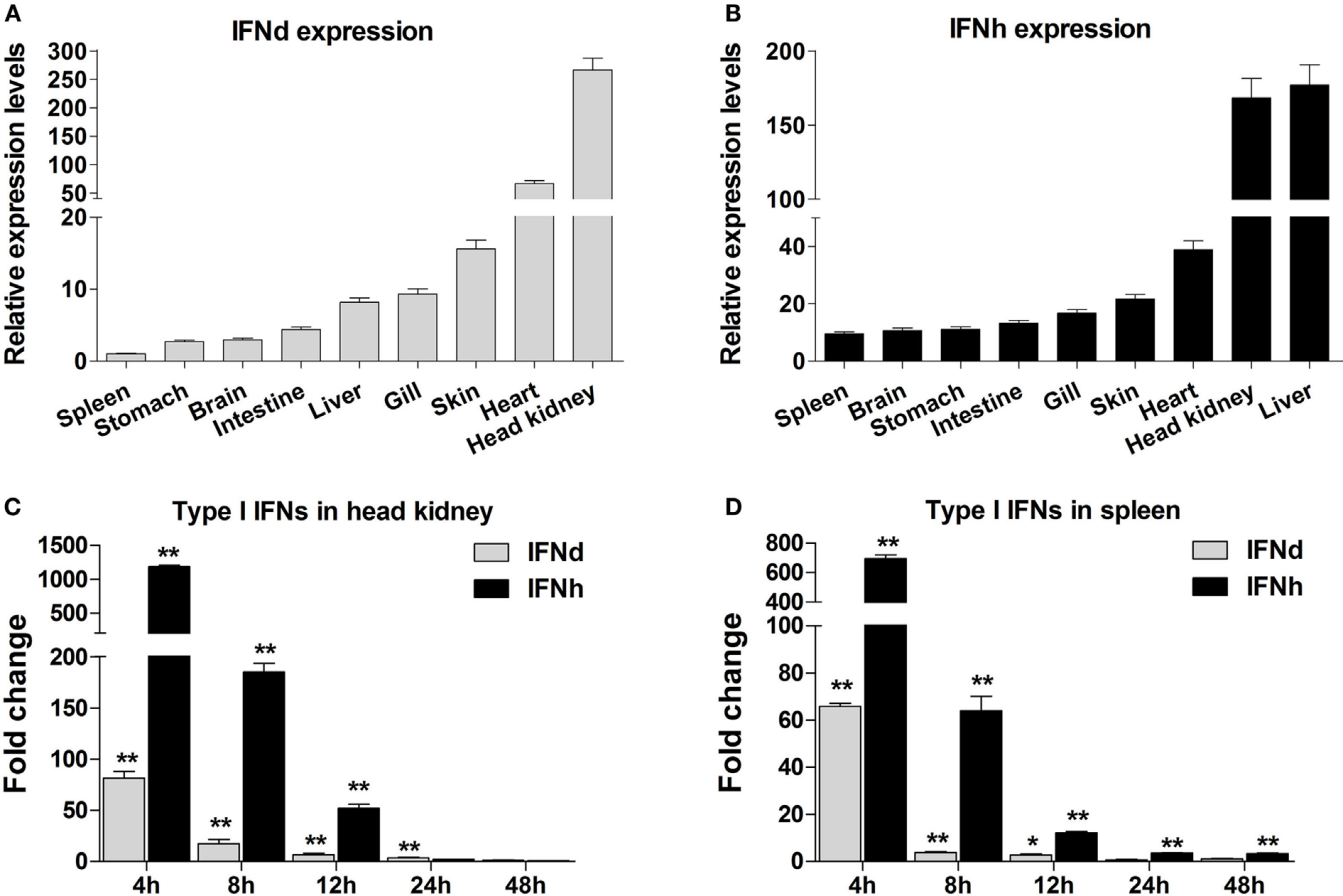
**Expression analysis of large yellow croaker IFNd and IFNh**. **(A,B)** Tissue expression profile of IFNd **(A)** and IFNh **(B)** genes. Total RNA was extracted from various tissues of five healthy fish and used for real-time PCR analysis. The expression levels of IFN genes were normalized by β-actin using the 2^−ΔΔCT^ method and expressed as the ratio of the IFNd expression levels in the spleen. The tissues were ordered according to the relative expression levels from the lowest to the highest. **(C,D)** Expression modulation of IFNd and IFNh genes in the head kidney **(C)** and spleen **(D)** after poly(I:C) induction. Each fish was intraperitoneally injected with 0.2 mg poly(I:C)/100 g fish or PBS (as a control), and head kidney and spleen tissues were collected from five fish in both groups at different time points postinjection for real-time PCR analysis. The expression levels of IFN genes were normalized by β-actin and the normalized expression levels compared between the poly(I:C)-injected fish and the PBS-injected fish (defined as 1) to obtain the relative fold changes at different time points. Error bars represent the standard error of the mean (± SEM) of three repeated experiments. *p < 0.05; **p < 0.01. The data were analyzed by two-tailed Student’s *t*-test.

The lyc IFNd and IFNh were constitutively expressed in all tissues analyzed, with varied expression levels detected. For example, the IFNd and IFNh were most highly expressed in the head kidney and liver, respectively (Figures 2A,B). Administration of poly(I:C) by intraperitoneal injection resulted in significant induction of IFNd and IFNh expression in head kidney and spleen, with the highest increases at 4 h for both genes (Figures 2C,D). Notably, the IFNh was more responsive than IFNd, showing remarkable increases of 1185- and 695-fold in head kidney and spleen, respectively (Figures 2C,D).

### Antiviral Activity of lyc IFNs

**Figure 3:**
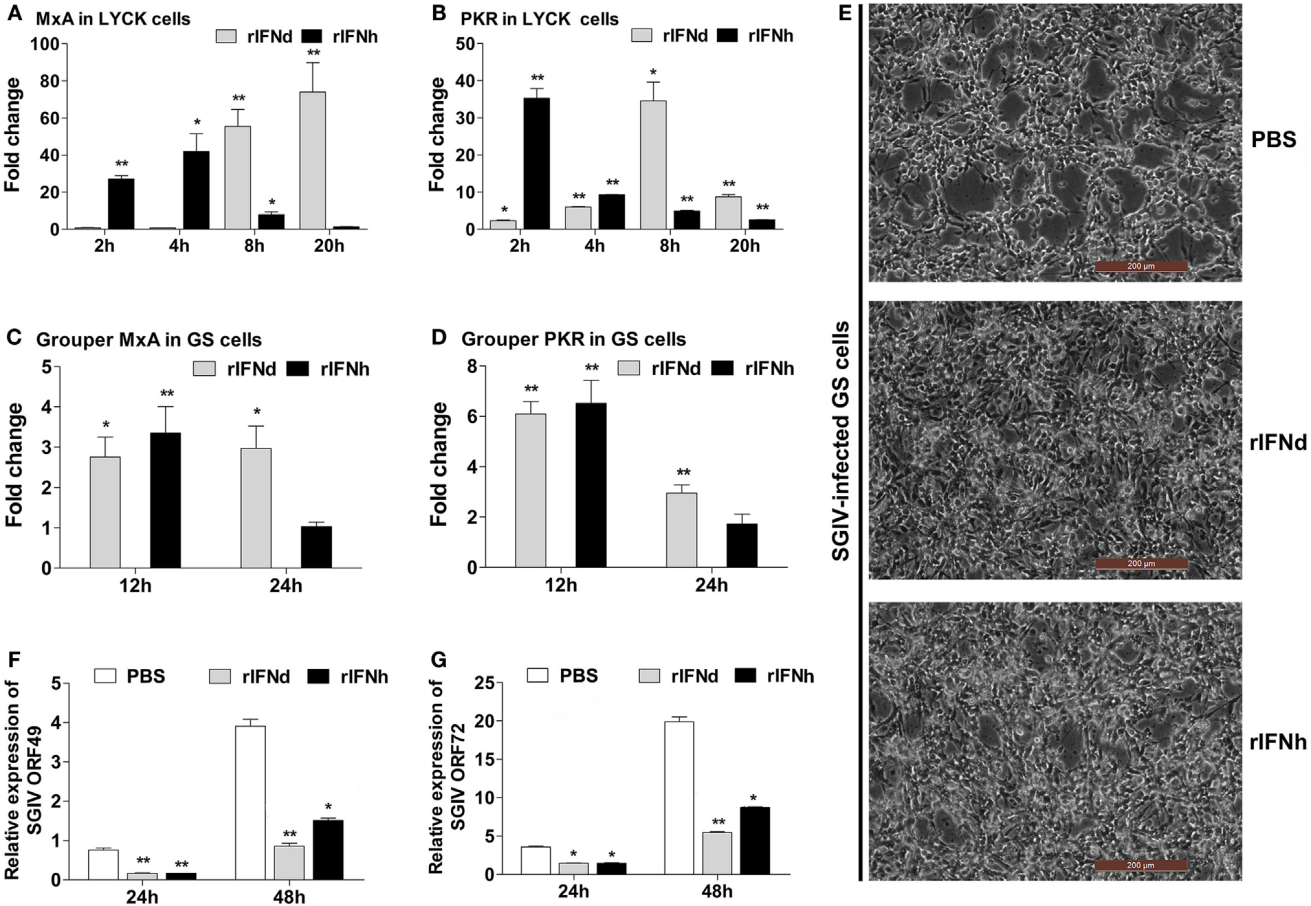
**Antiviral activities of recombinant large yellow croaker IFNd and IFNh**. **(A,B)** Induction of large yellow croaker MxA and PKR gene expression by rIFNs in LYCK cells. LYCK cells were plated in 6-well plates (1 × 10^6^ cells/well) and then treated with rIFNs at a final concentration of 50 ng/ml or PBS (control). LYCK cells were sampled at the indicated time points and used for real-time PCR analysis of MxA and PKR gene expression. The relative expression was normalized to the expression of β-actin, and fold induction was calculated by comparing the relative gene expression in rIFN-treated cells with that in PBS-treated cells (defined as 1) at the same time point. **(C,D)** Induction of grouper MxA and PKR gene expression by rIFNs in GS cells. The experiments were performed as described above. **(E)** GS cells were pretreated with rIFNs at a final concentration of 50 ng/ml or PBS (control) for 2 h; then, the cells were infected with SGIV at MOI 2. At 24 h postinfection, GS cells were observed for CPE using microscopy. **(F,G)** At 24 h and 48 h postinfection, the expression levels of SGIV ORF049 **(F)** and ORF072 **(G)** genes were detected by real-time PCR, and normalized to that of *Ec*β-actin. All data were obtained from three independent experiments with each performed in triplicate. Error bars represent the standard error of the mean (±SEM) of three independent experiments. *p < 0.05; **p < 0.01. The data were analyzed by two-tailed Student’s *t*-test.

To investigate whether lyc IFNs were able to induce the expression of antiviral genes, the LYCK cells were treated with rIFNd or rIFNh produced in HEK293T cells. Unsurprisingly, the two major antiviral genes, MxA and PKR, were significantly increased by the treatment of rIFNs. Interestingly, rIFNh gave rise to a more rapid activation of MxA and PKR, than the rIFNd (Figures 3A,B).

The antiviral activity of recombinant lyc IFNd and IFNh was examined using a cell line (GS) derived from orange-spotted grouper, where an infection model was already established (28, 33, 36). When stimulated with rIFNd or rIFNh for 24 h, the GS cells exhibited significant induction of MxA and PKR expression, confirming the cross-activity in the GS cells (Figures 3C,D). Subsequently, the GS cells were used for assessing the antiviral activity of recombinant lyc IFNd and IFNh. Pre-treatment with rIFNd and rIFNh 2 h prior to SGIV infection resulted in significant inhibition of CPE compared with the control cells (Figure 3E), indicating that lyc IFNd and IFNh were able to provide enhanced protection of GS cells against SGIV infection. This is supported by the obviously reduced expression of viral genes in the rIFN-treated GS cells (Figures 3F,G). These results, thus, indicated that both lyc IFNd and IFNh exhibited antiviral activity against SGIV in GS cells.

### Activation of the IFN Response by lyc IFNd and IFNh

**Figure 4:**
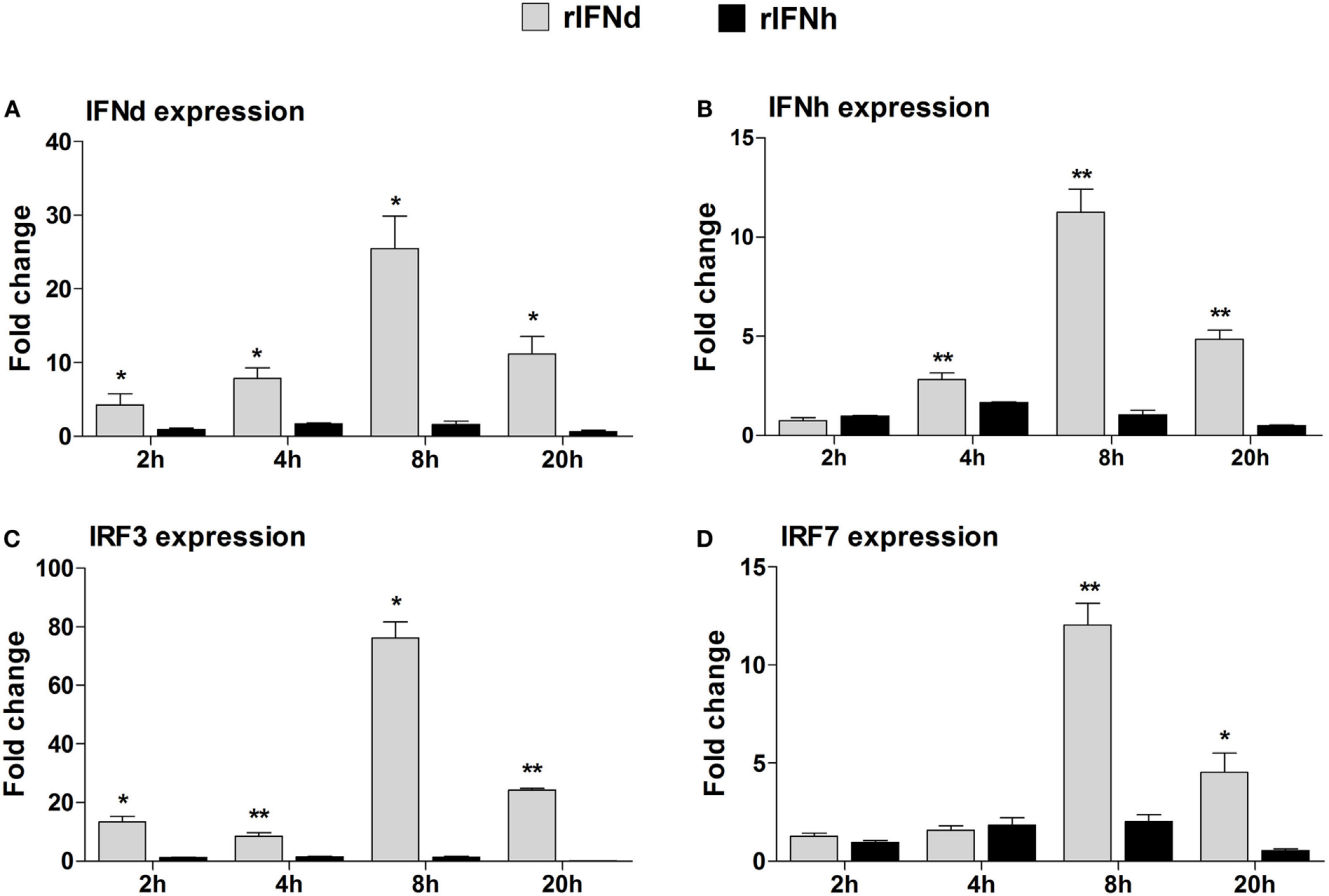
**Modulation of IFN-responsive genes by recombinant large yellow croaker IFNs in LYCK cells**. LYCKs were plated in 6-well plates (1 × 10^6^ cells/well) and then treated with rIFNd or rIFNh at a final concentration of 50 ng/ml or PBS. LYCK cells were sampled at the indicated time points and used for real-time PCR analysis of large yellow croaker IFNd **(A)**, IFNh **(B)**, IRF3 **(C)**, and IRF7 **(D)** gene expression. The relative expression levels of these genes were normalized by β-actin. Fold change of gene expression level was obtained by comparing the normalized gene expression level in rIFN-treated cells with that in PBS-treated cells (defined as 1) at the same time point. All data were obtained from three independent experiments with each performed in triplicate. Error bars represent the standard error of the mean (±SEM) of three independent experiments. *p < 0.05; **p < 0.01. The data were analyzed by two-tailed Student’s *t*-test.

To investigate whether lyc IFNd and IFNh were able to activate the IFN responses, the LYCK cells were stimulated with the recombinant lyc IFNs, and expression levels of lyc IFNd and IFNh were analyzed at 2, 4, 8, and 20 h postinduction. Surprisingly, the lyc IFNd significantly upregulated expression of both lyc IFNd and IFNh genes (Figures 4A,B). Furthermore, the transcript levels of lyc IRF3 and IRF7 were significantly increased by lyc IFNd treatment, with a greater increase of IRF3 transcripts than that of IRF7 transcripts (Figures 4C,D). In contrast, the lyc IFNh had no effect on the expression of lyc IFNd, IFNh, IRF3, and IRF7 (Figure 4).

**Figure 5:**
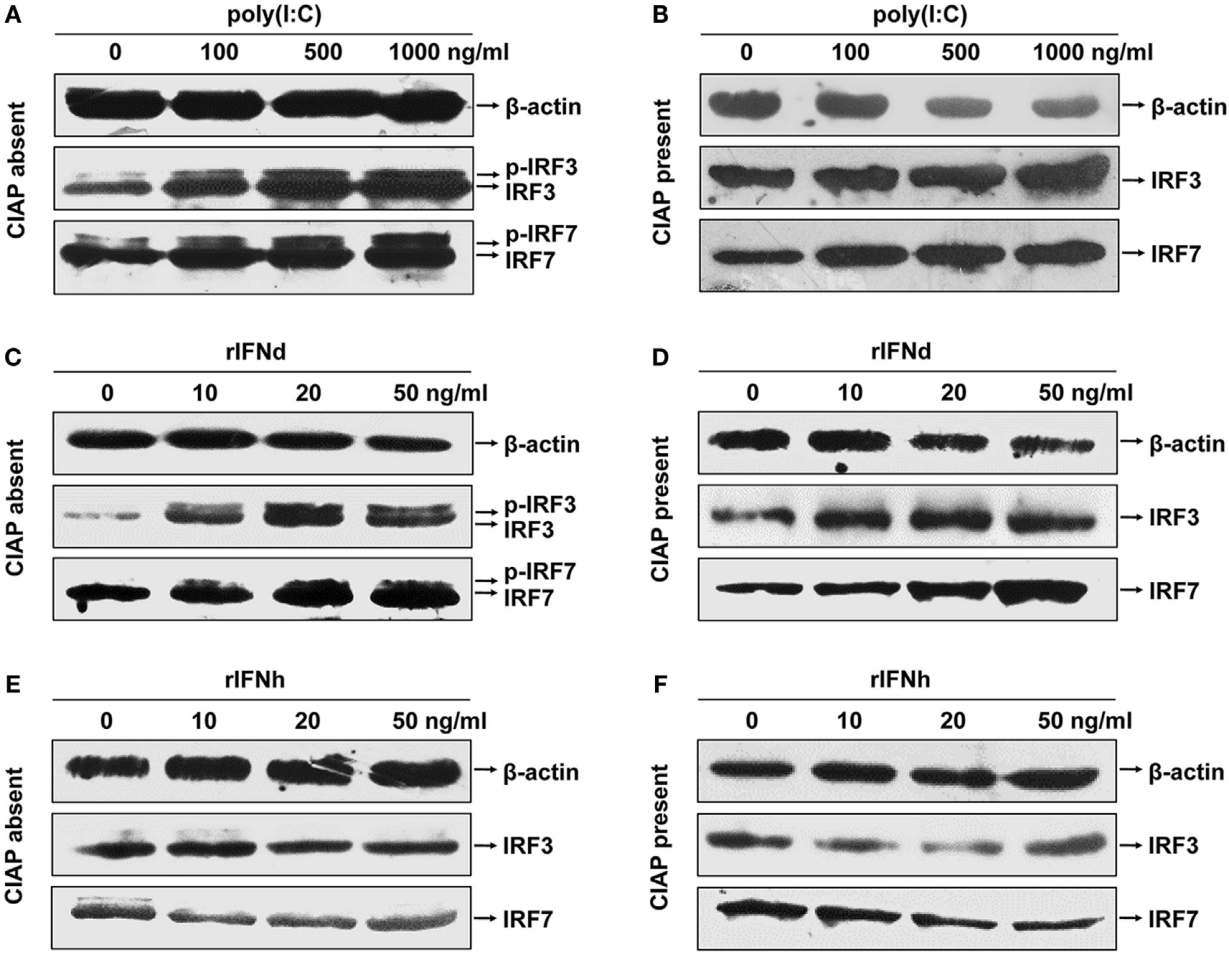
**Activation of large yellow croaker IRF3 and IRF7 in response to poly(I:C) and rIFNs**. LYCK cells were in 6-cm culture dishes (2.5 × 10^6^ cells/dish) overnight and then treated with poly(I:C) **(A,B)**, rIFNd **(C,D)**, and rIFNh **(E,F)** at a range of doses as indicated for 12 h. LYCK cell extracts were incubated with or without 20 U of calf intestinal alkaline phosphatase (CIAP) each sample for 30 min and then used to detect the induction and phosphorylation of IRF3 and IRF7 proteins by Western blotting analysis.

Next, we examined the phosphorylation of lyc IRF3 and IRF7 in the LYCK cells, following treatment with poly(I:C) and rIFNs. For this, polyclonal anti-IRF3 and anti-IRF7 Abs were generated and verified to specifically recognize their corresponding proteins expressed in HEK293T cells by Western blotting, thus excluding the possibility of cross-recognition between these two Abs (Figure S3C in Supplementary Material). In the LYCK cells treated with poly(I:C), both unphosphorylated and phosphorylated lyc IRF3 and IRF7 proteins were increased (Figures 5A,B). Similar effects were observed in the cells stimulated with rIFNd (Figures 5C,D). However, the rIFNh did not alter the levels of unphosphorylated and phosphorylated IRF3 and IRF7 (Figures 5E,F). These results suggest that the lyc IFNd, but not IFNh, was involved in the activation of IRF3 and IRF7, leading to induced IFN expression.

### Promoter Analysis of IFNd and IFNh Regulation

**Figure 6:**
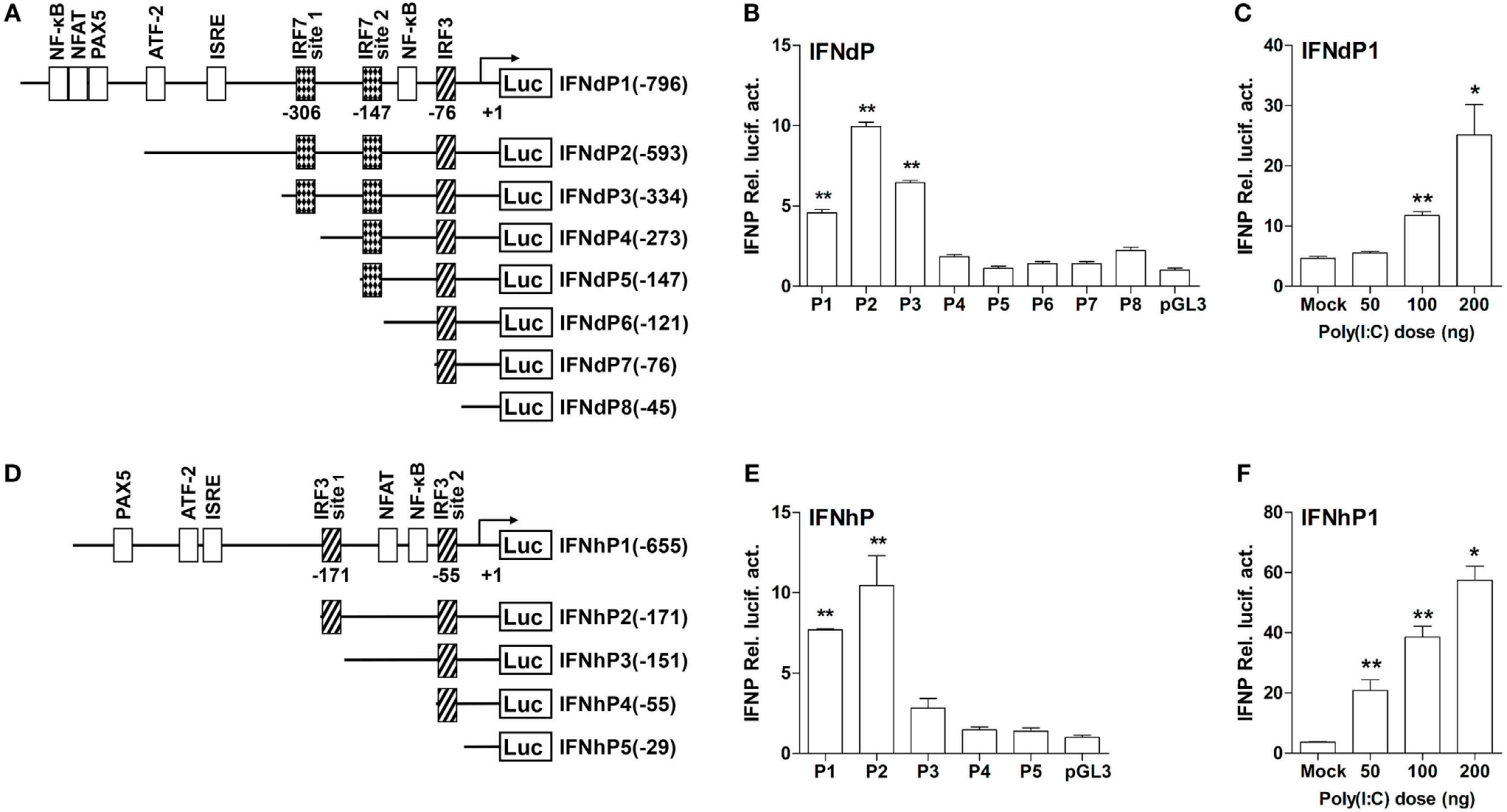
**The structure and transcriptional activity of large yellow croaker IFN promoters**. **(A,D)** Schematic representation of IFNd **(A)** and IFNh **(D)** promoters and a series of deletion constructs. **(B,E)** Transcriptional activity of IFN promoters. EPC cells (5 × 10^4^/well) were seeded in 96-well plates overnight and cotransfected with 100 ng of pGL3-IFNdP plasmid **(B)** or pGL3-IFNhP plasmid **(E)** and 2 ng of pRL-TK using the Fugene^®^ HD transfection reagent. Transcript levels were determined by real-time PCR. **(C,F)** Induction of large yellow croaker IFN promoter activity by poly(I:C). EPC cells (5 × 10^4^/well) were seeded in 96-well plates overnight and cotransfected with 50 ng of pGL3-IFNdP plasmid **(C)** or pGL3-IFNhP plasmid **(F)**, poly(I:C) (as indicated doses), and 1 ng of pRL-TK using the Fugene^®^ HD transfection reagent. After 48 h of transfection, the cells were harvested for detection of luciferase activity. All data were obtained from three independent experiments with three replicates in each experiment. Error bars represent ±SEM of three independent experiments. *p < 0.05; **p < 0.01. The data were analyzed by two-tailed Student’s *t*-test.

To understand why the two IFNs elicited distinct activities in induction of the IFN responses, the 1.2 kb 5′-flanking regions of lyc IFNd and IFNh gene promoter were analyzed to search for putative binding sites of transcription factors, including IRF3 and IRF7. As shown in Figure 6, the predicted binding sites for transcription factors were mainly located within 796 bp upstream of the transcription start site of IFNd promoter and 655 bp upstream of that of IFNh promoter. The lyc IFNd promoter contained one predicted IRF3 binding site and two IRF7 binding sites, while the IFNh promoter only had two predicted IRF3 binding sites. Binding sites for other transcription factors, such as the NF-κB, ATF-2, and PAX5 and NFAT families, were also predicted (Figures 6A,D). Luciferase assays further showed that both full-length IFNd and IFNh promoters (IFNdP1 and IFNhP1) had the ability to initiate the transcription of the luciferase reporter gene (Figures 6B,E) and that their expression could be enhanced by poly(I:C) treatment (Figures 6C,F).

To determine the active IRF binding sites, several different truncated mutants of IFNd and IFNh promoters were constructed. The IFNdP2 construct, containing two IRF7 binding sites (IRF7 site 1 at −306 bp and IRF7 site 2 at −147 bp) and an IRF3 binding site at −76 bp, was found to have the maximal transcriptional activity. However, after deleting the IRF7 site 1 (−306 bp) on IFNdP3, the promoter activity of this construct was largely reduced (Figure 6B), suggesting that the IRF7 site 1 was important for initiating the IFNd expression. IFNdP6 only containing the IRF3 binding site (−76 bp) exhibited no activity as well (Figure 6B). Similarly, the IFNhP2 construct, containing a distant IRF3 binding site (IRF3 site 1, −171 bp) and a proximal site (IRF3 site 2, −55 bp), showed the maximal transcriptional activity relative to the other constructs, and the deletion of the IRF3 site 1 (−171 bp) resulted in a significantly reduced promoter activity, suggesting that the IRF3 site 1 was essential for the IFNh promoter activity (Figure 6E). In contrast, the constructs only containing the IRF3 site 2 (−55 bp) did not induce luciferase activity, suggesting that this IRF3 site was not required for triggering the IFNh expression (Figure 6E).

**Figure 7:**
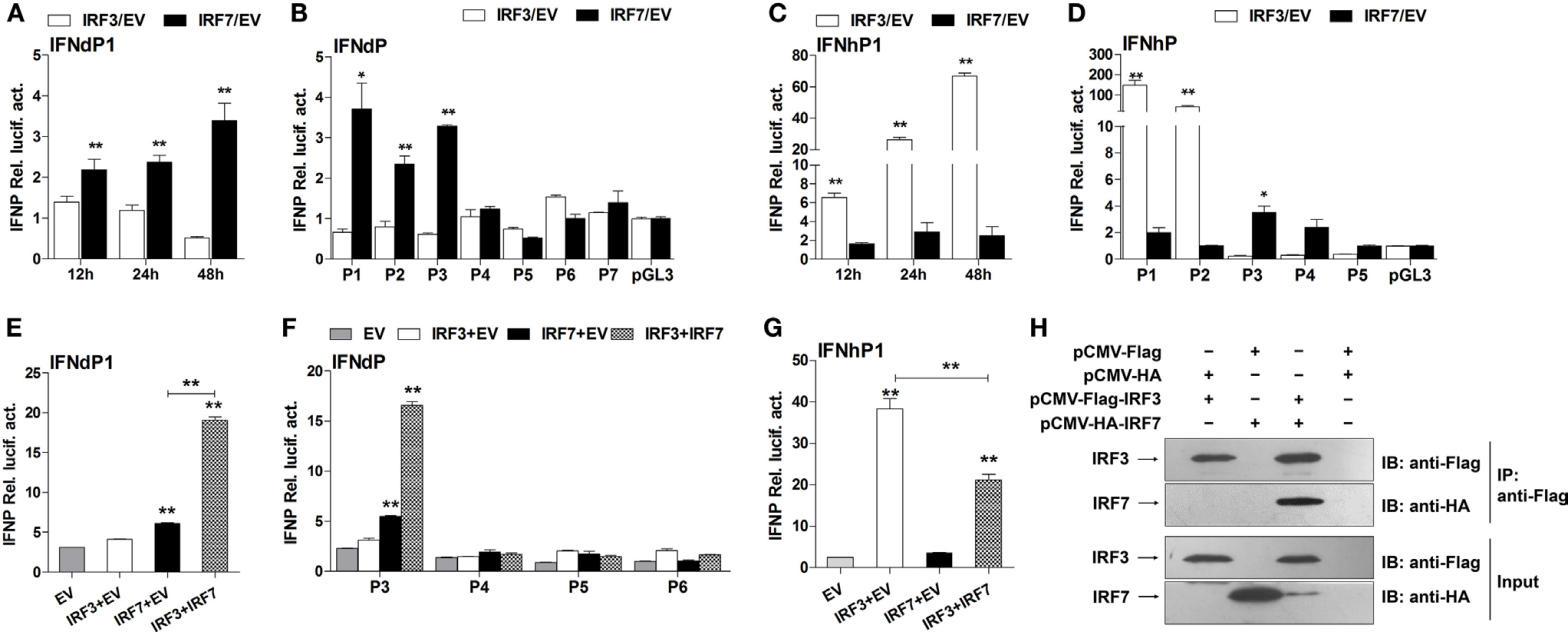
**Effects of IRF3 and IRF7 on induction of IFN promoter activity**. **(A)** Full-length IFNd promoter (IFNdP1), **(B)** the IFNdP deletion constructs, **(C)** full-length IFNh promoter (IFNhP1), **(D)** the IFNhP deletion constructs. EPC cells (5 × 10^4^/well) were seeded in 96-well plates overnight and cotransfected with 50 ng of pGL3-IFNP plasmids, 50 ng of pCMV-HA-IRF3 or -IRF7, and 1 ng of pRL-TK. **(E–G)** Cooperative effect of IRF3 and IRF7 on IFN promoter. EPC cells were seeded in 96-well plates overnight and cotransfected with 50 ng of IFNdP1 **(E)**, the IFNdP deletion constructs **(F)** or IFNhP1 **(G)**, the indicated expression constructs (100 ng total at a ratio of 1:1), and 1 ng of pRL-TK. After 48 h of transfection, the cells were harvested for detection of luciferase activity. The empty vectors (EV) were used as controls. All data were obtained from three independent experiments with three replicates in each experiment. Error bars represent ±SEM. *p < 0.05; **p < 0.01. The data were analyzed by two-tailed Student’s *t*-test. **(H)** Interaction of large yellow croaker IRF3 and IRF7. Protein extracts from HEK293T cells cotransfected with plasmids expressing Flag-tagged IRF3 and HA-tagged IRF7 were immunoprecipitated with murine anti-Flag monoclonal antibody. Immunoprecipitated complexes (IP) and whole cell lysates (Input) were analyzed by immunoblot (IB) for IRF3 and IRF7 using antibodies against Flag and HA.

Further transfection experiments were performed to gain insights into the regulatory roles of IRF3 and IRF7 on IFNd and IFNh expression. First, we confirmed that the exogenous IRF3 and IRF7 were produced in the EPC cells when transfected with expression constructs of lyc IRF3 or IRF7 (Figure S3D in Supplementary Material). The full-length IFNd promoter (IFNdP1) could be activated by overexpression of IRF7 (3.4-fold, Figure 7A). The truncated constructs IFNdP2 and P3, which contained the IRF7 site 1 (−306 bp), also showed induced luciferase activity in IRF7-overexpressed cells, whereas the IFNdP4 construct without the IRF7 site 1 did not show any altered luciferase activity (Figure 7B), indicating that the IRF7 site 1, but not the IRF7 site 2, was important for the IFNd expression. However, overexpression of IRF3 did not affect the luciferase activity driven by all the IFNd promoter constructs (Figure 7B).

In contrast, overexpression of IRF3 significantly enhanced the luciferase activity yielded by the full-length IFNh promoter (IFNhP1) and IFNhP2 constructs, but not the IFNhP3 construct where the IRF3 site 1 (−171 bp) was absent (Figures 7C,D), confirming that the IRF3 site 1 was essential for the activation of IFNh. The luciferase activity of the IFNh promoter was also increased by overexpression of IRF7 (2.6-fold), but considerably lower than that by overexpression of IRF3 (66.9-fold, Figure 7C). These results indicate that IRF3 and IRF7 have specific roles in regulating IFNd and IFNh, respectively.

Interestingly, cotransfection of IRF3 and IRF7 plasmids yielded much higher luciferase activity of the IFNd promoter (19.1-fold) than that of IRF7 alone (6.1-fold, Figure 7E). Again, the synergistic effect of IRF3 and IRF7 appeared to involve the IRF7 site 1, since overexpression of IRF3 and IRF7 did not stimulate the luciferase activity of the IFNdP4, P5, and P6 constructs lacking the IRF7 site 1 (Figure 7F). However, overexpression of IRF3 and IRF7 did not increase IFNh promoter activity to a greater extent than IRF3 alone (Figure 7G). Co-immunoprecipitation of IRF3 and IRF7 in the transfected cells revealed that lyc IRF3 could interact with lyc IRF7, which facilitated the formation of the IRF3 and IRF7 heterodimer and was important to the enhanced effect (Figure 7H). These results reveal that the IRF3 and IRF7 have distinct roles in regulating fish IFN expression, controlling the expression of IFNd and IFNh, respectively, and interaction of IRF3 and IRF7 could further enhance the IFNd, but not IFNh expression.

### Binding of IRF3/IRF7 to IFN Promoters

**Figure 8:**
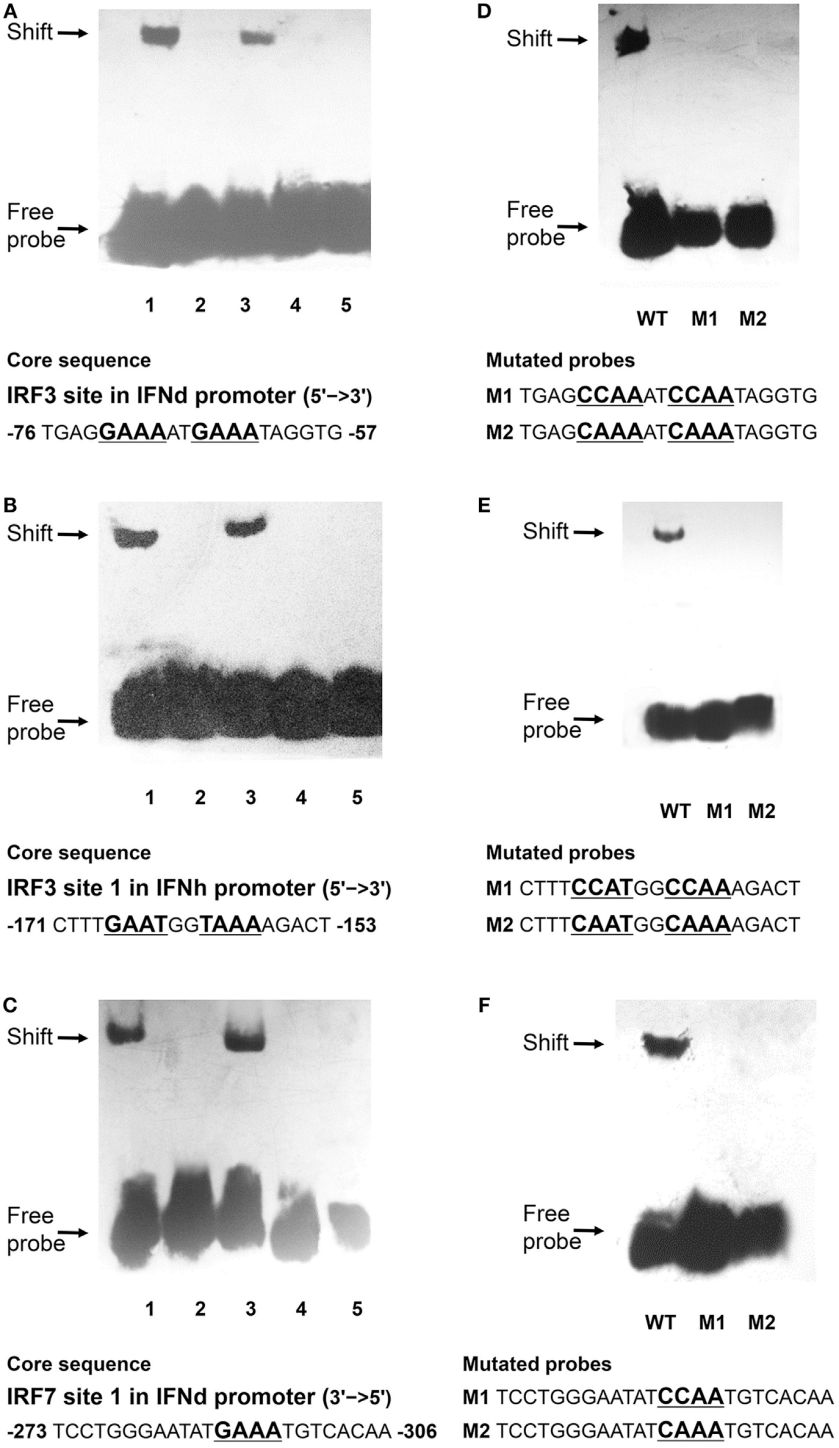
**Binding reactions of large yellow croaker IRFs and IFN promoters**. Biotin-labeled EMSA probes were incubated with lysates of HEK293T cells containing rIRF3 or rIRF7 proteins. **(A,B)** 1. IRF3 probes of IFNd **(A)** and IFNh **(B)** plus IRF3; 2. IRF3 probes only; 3. 100× unlabeled mutated probes plus IRF3; 4. 100× unlabeled wild-type probes plus IRF3; 5. IRF3 probes plus IRF7. **(C)** 1. IRF7 probes of IFNd plus IRF7; 2. IRF7 probes only; 3. 100× unlabeled mutated probes plus IRF7; 4. 100× unlabeled wild-type probes plus IRF7; 5. IRF7 probes plus IRF3. **(D)** Mutated IRF3 probes of IFNd and **(E)** mutated IRF3 probes of IFNh plus IRF3. **(F)** Mutated IRF7 probes of IFNd plus IRF7. After a 20 min incubation, the completed reactions were separated by electrophoresis on a 4% non-denaturing polyacrylamide gel for EMSA. WT, wild-type probes; M1 and M2: mutated probes.

The EMSA assay was performed to verify the IRF3/7 binding motifs in the IFN promoters characterized. The oligonucleotide probes were synthesized for the predicted IRF3 and IRF7 binding sites and incubated with cell lysates containing recombinant IRF3 or IRF7 *in vitro*. It is apparent that the rIRF3 was able to bind to the oligo probes of the predicted IRF3 binding sites in the IFNd promoter (−76 bp) and IFNh promoter (−171 bp). The formation of DNA–rIRF3 complex was specific since it could be blocked by excessive unlabeled control probes (100×) (Figures 8A,B). Furthermore, retardation of the IRF3 probes was not observed in the presence of rIRF7 protein, indicating that the IRF7 could not bind to the two IRF3 binding sites (Figures 8A,B). Subsequent mutations of the nucleotides in the IRF3 binding site resulted in dissociation of the DNAc–rIRF3 complex (Figures 8D,E). Similarly, the specific binding of rIRF7 and IRF7 binding site 1 (−306 bp) in the IFNd promoter was also confirmed (Figures 8C,F).

## Discussion

Teleost type I IFNs are classified into two groups based on the cysteine patterns in their mature peptides, with group I and II containing either two or four cysteines, respectively (3). Recent studies have shown that they can be further divided into six phylogenetic subgroups, including IFNa, b, c, d, e, and f (2). In the present study, we have identified two type I IFNs, IFNd and IFNh, in large yellow croaker, of which IFNh belongs to a novel subgroup (termed IFNh subgroup) of group I IFNs, based on the cysteine pattern and phylogenetic analyses (Figure 1, Figure S2 in Supplementary Material). The IFNh homologs were also discovered in zebra mbuna, Burton’s mouthbrooder, tilapia, and cichlid (Table S2 in Supplementary Material), suggesting that an IFN subgroup is commonly present in the Perciforme lineage in addition to the IFNd subgroup found previously (6–9). Thus, the Perciforme fishes possess at least two subgroups of group I IFNs, IFNd and IFNh. This is the first report that two subgroups of type I IFNs exist in Perciforme fishes, making the number of fish IFN subgroups to seven, the IFNa, d, e, and h belonging to the group I IFNs, while the IFNb, c, and f to the group II IFNs.

It is well known that teleosts have experienced a third round (3R) of whole-genome duplication (WGD) during their early evolution, and this WGD event is believed to contribute to the diversification of fish type I IFN family (37, 38). Salmonids are thought to have undergone an additional WGD event compared with other teleost species and possess six IFN subgroups, IFNa, d, and e subgroups of group I IFNs and IFNb, c, and f of group II IFNs (2, 39). Some, if not all of these, subgroups exist in primitive teleosts and may have been lost in certain teleost lineages during evolution. For instance, only three of the subgroups, IFNa, c, and d are present in cyprinids, such as zebrafish and carps (11, 40–42). However, the IFNh subgroup identified here cannot be assigned to any subgroups found in salmonids and cyprinids and, hence, represents a novel phylogenetic group. Considering that salmonids and cyprinids are relatively primitive teleosts, we suggest that the IFNh subgroup found in the Perciformes might have diverged more recently in evolution.

The expression patterns of type I IFNs in fish have been relatively well studied. In general, group I IFNs (IFNa, d, and e) appear to be constitutively expressed in most cell types and fish tissues and are inducible by viral RNA analogs or viral infection. In this study, the IFNd and IFNh (group I IFNs) were also shown to be constitutively expressed in all examined tissues, but with different levels of expression in tissues (Figures 2A,B). Upon poly(I:C) stimulation, the increase of IFNh transcripts was greatly higher than that of IFNd (1185-fold increases versus 82-fold increases in head kidney, and 695-fold increases versus 66-fold increases in spleen) (Figures 2C,D), indicating that the IFNd and IFNh are differentially modulated by poly(I:C) in head kidney and spleen. The results are in line with those observed in other fish species, such as trout, zebrafish, salmon, and medaka *O. latipes* (2, 10, 11, 43). Subsequent functional analyses demonstrate that the IFNh was more potent in triggering a rapid induction of the antiviral genes MxA and PKR than the IFNd (Figures 3A,B), suggesting that these two IFNs may play distinct roles in regulating early antiviral immunity.

Interferon regulatory factor 3 and IRF7 are master transcriptional factors that regulate type I IFN gene expression (44, 45). Mammalian IRF3 is constitutively expressed in most cell types and cannot be induced by IFN or viral analogs at the transcriptional level, whereas fish IRF3, on the contrary, has been confirmed to be a typical ISG (20, 46–49). The likely cause of this discrepancy may be explained by the presence of the IFN-stimulated response element (ISRE) motifs in the fish IRF3 promoters, including large yellow croaker (Figure S1C in Supplementary Material) (20, 46). In contrast to the IRF3, both mammalian and fish IRF7 can be induced by type I IFNs (48–51). Interestingly, the activation of both IRF3 and IRF7 in mammals requires virus-induced phosphorylation (17, 52), coordinating the appropriate initiation of IFN responses during virus infection. In the present study, not only were lyc IRF3 and IRF7 induced at the transcriptional level but also were phosphorylated by the treatment of IFNd or poly(I:C) (Figures 4C,D and 5C,D), suggesting distinct mechanisms for the activation of IRF3 and IRF7 between fish and mammals (17, 46, 52). The IFN-induced IRF3/IRF7 activation may be IFN type-specific, as the lyc IFNh failed to induce the expression and activation of IRF3 and IRF7 (Figures 5E,F).

In mammals, IRF3 functions mainly for the initiation of IFNβ gene expression, while IRF7 is more critical at the late stage for IFNα gene induction (16, 17). It is found that IRF7 governs the overall IFN responses and synergistically promotes the expression of type I IFNs with IRF3 (15, 52, 53). In fish, the IRF3 and IRF7 also display distinct roles in regulating type I IFN expression (19–22, 54). For example, the IRF7 binding sites are present in the promoter of IFNd, but not IFNh (Figure 6). The IRF7 could trigger IFNd expression by itself and induce much higher IFNd expression together with IRF3 (Figures 7A,E), in agreement with the previous observations in carp and zebrafish, where cooperation of IRF3 and IRF7 led to higher induction of IFN expression than IRF3 or IRF7 alone (54). In fact, lyc IRF3 and IRF7 could form heterodimer (Figure 7H), which may be transported into the nucleus and bind to the IFN promoter more effectively than the IRF3 or IRF7 homodimer alone. The EMSA assays further showed that both lyc IRF3 and IRF7 specifically bound to the IFNd promoter at the sites of −76 and −306, respectively (Figures 8A,C). Curiously, the lyc IFNh expression involves IRF3. Overexpression of IRF3 greatly enhanced the transcriptional activity of the IFNh promoter (Figure 7C), likely through binding to the IRF3 site 1 (−171) in the IFNh promoter (Figure 7D), as shown by the EMSA that IRF3, but not IRF7, specifically was involved in interaction with this motif (Figure 8B).

**Figure 9:**
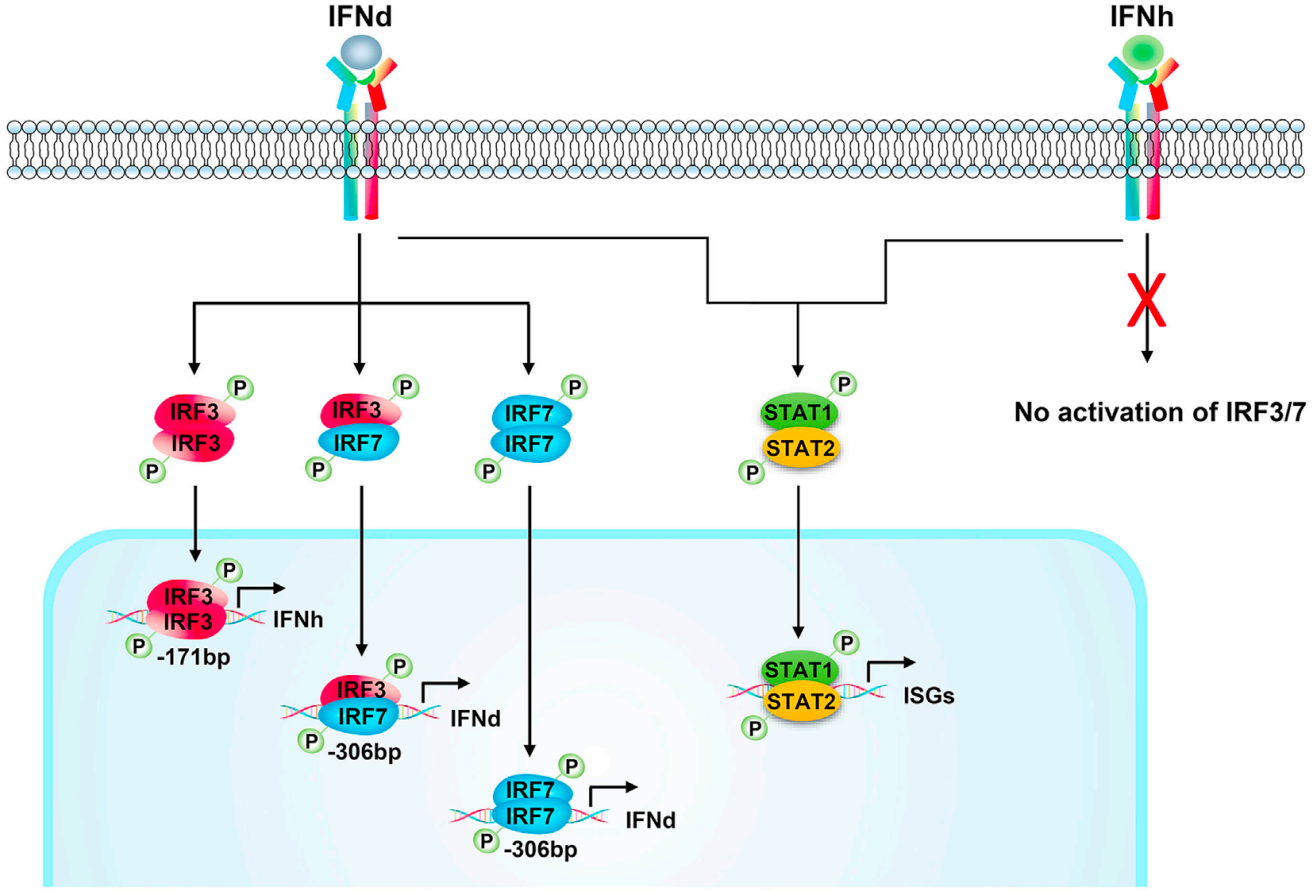
**Positive feedback regulation of type I IFN response mediated by IFNd-induced IRF3 and IRF7 activation in large yellow croaker**. The large yellow croaker IFNd induced the expression of itself and IFNh by activating IRF3 and IRF7. The dimeric form of IRF3 and IRF7 or IRF7 may be transported into the nucleus and bind to the corresponding sites in the IFNd promoter, thus upregulating the expression of IFNd. The activated IRF3 binds to the IRF3 site 1 (−171bp) present in the IFNh promoter and induces the expression of IFNh. In contrast, the large yellow croaker IFNh has no effect on the expression of itself and IFNd and expression and phosphorylation of IRF3 and IRF7. Both, IFNd and IFNh, are able to induce the expression of ISGs, including MxA and PKR, possibly through the Jak–Stat pathway.

In summary, we have identified two type I IFNs in large yellow croaker, one of which was assigned to a novel subgroup of fish group I IFNs (IFNh). The two IFNs (lyc IFNd and IFNh) showed apparent differences in expression patterns and ability to induce antiviral genes. Only IFNd, but not IFNh, was able to activate phosphorylation of IRF3 and IRF7 and trigger the expression of itself and IFNh. Furthermore, the expression of IFNd can be enhanced by the synergistic effect of IRF3 and IRF7, and the IFNh expression mainly involves IRF3. Thus, a positive feedback regulation, which was mediated by IFNd-induced IRF3 and IRF7 activation, was proposed in lyc (Figure 9). This IFN-induced IRF3 and IRF7 activation may represent a unique mechanism regulating fish IFN responses (Figure 9), which differs from that in mammals (17, 46, 52). The results provide new insights into the regulation and function of fish type I IFNs and further reveal the complexity of the regulatory mechanisms.

## Author Contributions

YD performed most of the experiments, analyzed the data, and wrote the manuscript. XH prepared the GS cells and SGIV and performed the experiments for antiviral activities. JA helped with the first draft and performed the statistical analysis. XC designed the research, mentored all other authors, and wrote the final version of the paper.

## Conflict of Interest Statement

The authors declare that the research was conducted in the absence of any commercial or financial relationships that could be construed as a potential conflict of interest.

## Footnotes

^1^http://www.cbs.dtu.dk/services/SignalP

^2^http://www.ncbi.nlm.nih.gov/IEB/Research/Ostell/Spidey

^3^http://www.genomatix.de/
