# Supplementary material for: Identification of Two Subgroups of Type I IFNs in Perciforme Fish Large Yellow Croaker Larimichthys crocea Provides Novel Insights into Function and Regulation of Fish Type I IFNs
Source: Front Immunol. 2016 Sep 7;7:343. doi: 10.3389/fimmu.2016.00343 (PMC5013148; doi:10.3389/fimmu.2016.00343)
Supplement: Supplementary file 1 [file table_1.pdf]

Supplementary Table 1

Identification of Two Subgroups of Type I IFNs in Perciforme Fish Large Yellow Croaker *Larimichthys crocea* Provides Novel Insights into Function and Regulation of Fish Type I IFNs

Yang Ding*, Jingqun Ao, Xiaohong Huang

*** Correspondence:** Xinhua Chen: chenxinhua@tio.org.cn

Supplementary Table 1 | Oligonucleotide primers and probes used in this study.

Primer name	Nucleotide sequence (5′→3′)	Purpose
IFNd-F	ATGCTCAGCAGGATCTTGTTTGT	Gene cloning
IFNd-R	TTAGTTGGTGTTGCGACGGAGAT	
IFNh-F	ATGGTTAACTGGACCGGCGTG	
IFNh-R	TCAGTGCTGCCGTCCACTCG	
IRF3-F	ATGTCTCATTCTAAACCTCTGC	
IRF3-R	TCAGTACAGCTCCATCATCTCT	
IRF7-F	ATGCAAAGCCCTCCCAAGC	
IRF7-R	TCAATAAAGCTCAGCAGCCAGTG	
IFNd promoter-F	TGAAATGAGGATCCTCACACC	
IFNd promoter-R	GGACTGAGAGACAGGCACACA	
IFNh promoter-F	ATCTGATGGACGTAACGTAGAGA	
IFNh promoter-R	CCCACAGAGGACGAAGAGC	
IFNd-RF-Flag	GGAATTCCATGCAGATGGGTGGAT	Recombinant expression
IFNd-RR-Flag	CGGGATCCGTTGGTGTTGCGCA	
IFNh-RF-Flag	GGAATTCCATGTGATTGGCTCAGAC	
IFNh-RR-Flag	CGGGATCCGTGCTGCCGTCC	
IRF3-RF-Flag	GGAATTCGGATGTCTCATTCTAAACCTC	
IRF3-RR-Flag	CCCAAGCTTTCAGTACAGCTCCATCA	
IRF3-RF-HA	GGAATTCGGATGTCTCATTCTAAACC	
IRF3-RR-HA	GGGGTACCTCAGTACAGCTCCATCA	
IRF7-RF-HA	GGGGTACCATGCAAAGCCCTC	
IRF7-RR-HA	GCGGCCGCTCAATAAAGCTCA	
IRF3-DBD-F	GGAATTCATGTCTCATTCTAAACC	
IRF3-DBD-R	CCCAAGCTTAGCTTTTGATGCTGA	
IRF7-DBD-F	GGAATTCATGCAAAGCCCTC	
IRF7-DBD-R	CCCAAGCTTAGTGTTGACAATCTC	
IFNd-RT-F	CTTGTTTGTGTGCCTGTCTCTC	Real-time PCR
IFNd-RT-R	GTCCTCCACCTGAGCATCC	
IFNh-RT-F	GGACTGGACAGGAGTGTCGAG	
IFNh-RT-R	CAGGAAGCAGAGGTGAGGTTG	
IRF3-RT-F	GTAGCAGACAGCCCATAGAGC	
IRF3-RT-R	CCATTGAAGTCAGGACCACC	

IRF7-RT-F	TGGCTTATAGAGCAGGTGGAG	
IRF7-RT-R	AGGTTATTCAGAGCGCATCG	
MxA-RT-F	GATGCTATAAGCCTCACCACA	
MxA-RT-R	GTTGATAAATCCTGGCAGTTC	
PKR-RT-F	CTAAAAAGTCCTGGGCAAGAA	
PKR-RT-R	TCGGTTTCTCAGAGTAGTTGGA	
β-actin-RT-F	GACCTGACAGACTACCTCATG	
β-actin-RT-R	AGTTGAAGGTGGTCTCGTGGA	
*Ec*MxA-RT-F	CGAAAGTACCGTGGACGAGAA	
*Ec*MxA-RT-R	TGTTTGATCTGCTCCTTGACCAT	
*Ec*PKR-RT-F	AAATGCCTTGAAATGCTTGTTG	
*Ec*PKR-RT-R	CTTCCTTGACAGTCCTTCCCTC	
RT-049-F	ATGTACGTATACCCCGCAAT	
RT-049-R	TCATTTTTTTTGCCTAA	
RT-072-F	GCACGCTTCTCTCACCTTCA	
RT-072-R	AACGGCAACGGGAGCACTA	
*Ec*actin-RT-F	TACGAGCTGCCTGACGGACA	
*Ec*actin-RT-R	GGCTGTGATCTCCTTCTGCA	
IFNd-PR-F	CCGCTCGAGTTCATGCTCAGGTAA	Promoter cloning
IFNd-PR-R	GGATCCCAGGAGTCAGGAGGGTCC	
IFNh-PR-F	CCGCTCGAGTCCTCATTCATCACC	
IFNh-PR-R	GGATCCGCACTGTACAGACTGAGAGA	
IFNdP(-76~-57)-F	TGAGGAAAATGAAATAGGTG	EMSA probe
IFNdP(-76~-57)-R	CACCTATTTCATTTTCCTCA	
IFNdP(-306~-273)-F	TCCTGGAATATGAAATGTCACAA	
IFNdP(-306~-273)-R	TTGTGACATTTCATATTCCAGGA	
IFNhP(-171~-153)-F	CTTTGAATGGTAAAAGACT	
IFNhP(-171~-153)-R	AGTCTTTTACCATTCAAAG	
