# Supplementary material for: Identification of Two Subgroups of Type I IFNs in Perciforme Fish Large Yellow Croaker Larimichthys crocea Provides Novel Insights into Function and Regulation of Fish Type I IFNs
Source: Front Immunol. 2016 Sep 7;7:343. doi: 10.3389/fimmu.2016.00343 (PMC5013148; doi:10.3389/fimmu.2016.00343)
Supplement: Supplementary file 2 [file table_2.pdf]

Supplementary Table 2

Identification of Two Subgroups of Type I IFNs in Perciforme Fish Large Yellow Croaker *Larimichthys crocea* Provides Novel Insights into Function and Regulation of Fish Type I IFNs

Yang Ding*, Jingqun Ao, Xiaohong Huang

*** Correspondence:** Xinhua Chen: chenxinhua@tio.org.cn

Supplementary Table 2 | Comparison of identities (top right) and similarities (bottom left) of large yellow croaker IFNd and IFNh with other teleost type I IFNs.

	IFNd														IFNh					IFNa									
	1	2	3	4	5	6	7	8	9	10	11	12	13	14	15	16	17	18	19	20	21	22	23	24	25	26	27	28	29
1.*Lc*IFNd		82.8	82.3	73	69.2	68.1	66	58.6	55.7	54.5	54.1	45.7	45.2	34	27.7	26.3	25.8	25	23.1	31.1	31.1	26.7	31.7	29.2	23.9	27.9	30.6	31.2	31.7
2.*Dl*IFN1	91.4		90.9	70.8	73	67.2	65.6	58.8	58.4	58.1	56.2	41.7	40.6	33	29.1	24.9	25.3	22.4	21.9	31.4	31.4	28.9	31.2	29	26.3	28.2	31.6	35.5	37.1
3.*Sa*IFN1	90.9	95.2		71.5	69.9	66.1	67.2	57.2	55.4	57.1	55.4	41.5	40.4	33.3	30.9	25.7	26.1	23.3	22.6	31.4	31.4	28.6	31.2	30.2	25.3	28.2	31.8	31.6	33.2
4.*Ec*IFN1	85.9	85.9	84.9		60.3	65.4	57.4	54.3	50.8	48.9	50	39.9	41	28.4	27.5	24.9	25.4	23.1	22.5	33.3	33.3	27.6	32.1	28.8	24.4	29.4	31.4	34.4	35
5.*Po*IFN1	83.2	83.2	81.7	78.8		61.6	57.9	52.7	51.6	48.7	51.1	39.9	39.4	35.5	27.9	27	27.9	24.4	22.3	28.4	28.4	26.2	28.4	25.5	23.9	29.4	29.2	33	35.1
6.*Ga*IFN1	82.7	81.6	81.2	82	78.3		55.9	53.4	48.6	46.8	46.2	39	39	32.2	30.8	26.1	26.2	25	22.4	28.9	28.9	27.9	29.6	25.3	26.4	26.9	28	30.4	32.4
7.*On*IFN3	80.2	79.7	82.9	75.4	75.9	74.9		56.5	45.2	59.4	44.7	41.1	42.1	28	28.9	27	27.4	25.1	25.1	31.1	31.1	27.4	31.8	28.9	23.2	29.8	28.9	33.5	32.4
8.*Ol*IFN	73.1	74.7	72.6	71.5	71	75.8	74.9		44.6	49.5	42.5	38.7	39.8	30.7	31.9	28.3	29.2	26.8	24.3	32.5	32.5	32.1	31.1	32.1	23.2	27.2	32.5	31.9	33
9.*Tr*IFN1	72.4	76.8	73.7	70	75	72.8	67.4	65.6		43.5	66.7	40.1	38.9	26.2	25.5	26	25.9	25.1	23.1	29.1	29.1	29.5	28.6	30.9	22.6	25.1	28	31.7	31
10.*On*IFN1	70.5	72.1	72.6	69.5	65.8	66.8	75.3	67.9	60		41.4	31.6	31.6	28.1	30.1	23.6	24.1	22.9	21.4	28.6	28.6	30.9	28.8	28.6	24.5	28.1	32.3	32.5	33
11.*Tn*IFN	70.8	75.1	74.2	68.5	70.7	71.3	67.4	64	83.3	59.5		36.8	36.2	32.6	26.5	23.6	24.1	22.6	20.1	28.2	28.2	31.3	28.2	27.6	22.1	25.6	28.7	31.7	34.3
12.*Om*IFNd1	64.9	62.2	61.3	65.7	64.1	63.5	62.6	59.7	61.7	51.6	61.2		91.6	30.2	27.4	27.3	26.3	23.8	23.1	33.7	33.7	33	34	31.9	22.3	26.8	33.9	36.5	38.7
13.*Ss*IFNd	65.9	61.6	61.3	63.5	63	61.8	63.1	61.8	61.7	52.1	59.6	97.8		30.9	27.3	25.8	27.4	23.3	22.7	34.4	34.4	31.9	34	32.3	22.3	25.8	31.2	34.8	36.3
14.*Dr*IFN4	55.1	50.8	50.5	49.4	54.3	52.8	50.8	47.8	53.3	44.7	52.8	52.8	56.2		27.3	23.3	24.3	20.4	19.9	22.7	22.7	23.5	22.6	22.3	23.2	23.2	24.5	28.2	30
15.*Lc*IFNh	48.4	49.5	51.1	46.3	50.5	50.5	52.1	52.6	45.3	52.1	43.2	47.4	47.4	43.7		55.2	53.1	52.2	48.4	30.5	30.5	30	28.7	29.4	21.2	29	28.4	33	35.1
16.*Hb*IFNal3	46.5	45	45	44.6	48.5	44.6	48	47.5	43.1	45	39.6	43.1	44.6	42.1	67.3		92.2	90.6	80.9	26.9	26.9	23.1	26.3	23.1	17.1	19.8	23	27.7	28.6
17.*On*IFNal3	46.6	44.7	45.1	43.2	48.5	42.7	48.1	45.6	42.2	44.2	38.8	41.7	44.2	40.8	66.5	96.1		85.4	77.1	25.9	25.9	21.8	25.3	22	16.8	20.4	23	27.1	28.1
18.*Mz*IFNal3	46.2	44.6	45.2	44.6	48.9	44.6	47.1	48.4	44.6	44.2	43.5	42.5	44.1	41.4	66.8	92.1	88.8		78	23.7	23.7	23.4	23.1	22	17.4	19.6	20.6	25.2	25.2
19.*Pn*IFNal3	39	39.4	39.8	38.6	41.1	38.6	43.2	42.4	38.1	39.4	35.6	36.4	38.6	37.3	57.2	83.5	82.6	77.5		23.1	23.1	22.6	22.2	22.2	14.8	18.5	20.2	23.2	23.6
20.*Ca*IFN	53	50.3	51.6	57.2	52.7	52.8	53.5	51.6	51.7	51.6	50	54.4	53.9	46.7	53.7	48	47.6	48.9	42.8		100	69.9	95.1	57.9	34.7	40.2	67.7	39.1	44
21.*Ci*IFN	53	50.3	51.6	57.2	52.7	52.8	53.5	51.6	51.7	51.6	50	54.4	53.9	46.7	53.7	48	47.6	48.9	42.8	100		69.9	95.1	57.9	34.7	40.2	67.7	39.1	44
22.*Cc*IFN	48.1	49.7	48.4	52.5	52.2	56.8		52.2	49.2	48.9	46.4	55.7	55.2	46.4	52.6	44.1	45.1	47.8	41.1	83.6	83.6		69.9	62.2	32.8	38.1	65.2	41.4	43.5
23.*Mp*IFN	55.7	53	52.7	56.2	52.4	54.6	55.6	53.2	51.4	53.2	49.2	53.5	53	46.5	53.2	48.5	48.1	49.5	42.8	95.7	95.7	84.9		58.5	33.7	39.6	67.9	38.3	43.1
24.*Cm*IFN	46.5	46.5	45.7	49.7	49.5	48.6	50.3	50	48.1	44.7	43.7	51.9	53.6	44.3	49.5	42.1	43.2	45.2	40.3	70.5	70.5	77	74.1		31.4	35.9	54.2	36.1	38.2
25.*Ip*IFN	46.5	46.5	45.2	46.6	46.7	50	43.9	41.9	45	48.4	47.2	46.1	46.6	42.2	43.2	41.6	40.8	43	35.6	55	55	53.6	53	49.7		62.4	35.7	33.3	35.4
26.*Ip*IFN2	50.5	50.5	50	49.5	48.5	47.9	50	44.3	47.4	52.1	43.8	50.5	49.5	43.8	49	44.6	44.2	43.3	40.3	62.4	62.4	57.7	62.4	50.5	73.2		38.3	33.3	36.9
27.*Dr*IFN1	57.3	55.7	55.4	58.6	54.3	56.4	54	53.2	51.4	58.4	50.3	58	56.9	45.9	53.2	50	48.5	49.5	43.6	81.8	81.8	81.4	80.5	74.9	57.5	59.8		37.9	39.9
28.*Om*IFNa1	54.1	55.7	53.8	55.1	56	57.3	56.1	54.3	56.7	56.8	57.4	59.6	60.1	51.4	55.8	51.5	49.5	50.5	44.5	59.4	59.4	59	57.3	50.8	52	50	56.9		89.7
29.*Ss*IFNa1	54.6	56.8	54.8	56.2	58.7	56.2	55.1	53.8	57.2	54.7	58	61.2	58.4	53.7	57.9	50.5	48.5	50	43.6	63.3	63.3	61.2	61.1	53	54.3	52.6	60.8	94.3	
30.*Om*IFNe1	47.8	52.2	51.6	48.4	52.2	47.8	49.2	54.3	51.1	47.9	49.5	51.1	51.6	45.7	47.4	40.6	40.8	39.8	35.6	55.9	55.9	52.7	54.3	48.9	46.2	49.5	55.4	51.6	53.2
31.*Om*IFNe2	43.9	48	47.5	44.9	49.5	43.4	46.5	51	46.5	47.5	47.5	47.5	48	44.4	46.5	40.6	42.2	37.9	35.6	52	52	49.5	50.5	45.5	42.4	48	51.5	48	49.5
32.*Om*IFNe3	43.9	48.5	48	45.5	49	43.9	45.5	51.5	46.5	47.5	47	48.5	49	44.4	46.5	41.1	42.2	38.9	36	52.5	52.5	50	51	46	43.9	48.5	52	50	51.5
33.*Om*IFNe4	46.1	49.2	49.7	47.7	49.7	46.1	46.1	52.8	48.7	48.2	46.6	49.7	50.3	46.1	47.2	39.1	40.3	36.3	33.9	51.3	51.3	50.3	50.3	48.2	43	47.4	50.8	48.7	50.3
34.*Om*IFNe5	48.9	49.5	48.9	47.3	51.1	47.3	46.5	52.7	51.6	45.3	48.9	52.2	52.2	43.5	50	42.6	40.3	40.9	34.3	53.8	53.8	50	53.8	47.3	44.1	49	52.2	50.5	50.5
35.*Om*IFNe6	49.5	50	49.5	47.3	51.1	47.8	47.1	53.2	52.2	44.7	48.9	52.7	52.7	45.7	50	42.6	42.2	43	35.6	53.8	53.8	50	53.8	47.3	44.1	49	52.2	51.1	50.5
36.*Om*IFNe7	48.9	50.5	50	46.8	50	46.8	44.9	51.6	50.5	44.2	48.9	52.2	52.7	44.1	49.5	44.1	41.3	44.1	36.4	53.8	53.8	48.9	53.8	47.3	46.8	49.5	51.6	50	50
37.*Om*IFNb1	51.4	49.7	47.3	45.9	53.3	53	50.3	49.5	53	46.8	50.3	55.2	54.6	47	49.5	46	46.1	43.5	42.4	47.5	47.5	47.5	47.6	48.1	39.3	42.3	52.5	51.4	51.9
38.*Om*IFNb2	50.3	48.6	46.2	44.8	52.7	51.9	50.3	48.9	51.9	45.8	49.7	54.1	54.6	47	48.4	46.5	46.6	44.1	42.8	47.5	47.5	47	47.6	47	39.3	41.8	51.9	50.8	51.4
39.*Om*IFNb3	51.4	49.7	47.3	46.4	53.3	53	50.3	49.5	53	46.8	50.3	55.2	54.6	47	49.5	46	46.1	43.5	42.4	48.1	48.1	48.1	48.1	48.6	39.9	42.8	53	50.3	50.8
40.*Om*IFNb4	50.8	49.2	46.8	48.1	53.8	53	50.3	49.5	53.6	46.3	50.8	55.7	55.2	46.4	50	46	45.6	43.5	42.4	47	47	47.5	47.6	48.6	39.3	42.3	51.9	51.9	52.5
41.*Om*IFNb5	50.8	49.2	46.8	48.1	53.8	53	50.3	49.5	53.6	46.3	50.8	55.7	55.2	46.4	50	46	45.6	43.5	42.4	47	47	47.5	47.6	48.6	39.3	42.3	51.9	51.9	52.5
42.*Ss*IFNb1	50.3	50.3	46.8	46.4	53.3	52.5	49.2	48.9	51.9	47.4	50.8	53	53	45.4	49.5	47.5	46.6	44.6	40.7	47.5	47.5	48.1	47.6	47.5	38.3	41.8	51.4	50.8	50.3
43.*Ss*IFNb2	50.3	49.7	46.2	45.9	53.3	52.5	49.2	48.4	51.9	46.8	50.3	54.1	53.6	47	47.9	47	46.1	44.1	40.7	48.1	48.1	48.1	48.1	48.6	39.3	42.3	53	51.4	51.9
44.*Ss*IFNb3	50.3	49.7	46.2	45.9	53.3	52.5	49.2	48.4	51.9	46.8	50.3	54.1	53.6	47	47.9	47	46.1	44.1	40.7	48.1	48.1	48.1	48.1	48.6	39.3	42.3	53	51.4	51.9
45.*Om*IFNc1	44.9	44.4	43.9	44.9	48.1	46.5	43.9	44.4	49.7	44.2	47.6	46	43.9	43.9	46.3	43.1	42.7	42.8	37.7	46.5	46.5	48.7	47.6	42.8	41.2	43.8	47.6	46.5	48.1
46.*Om*IFNc2	46	42.8	42.2	43.3	48.1	45.5	42.8	43.3	47.6	41.6	47.6	46.5	44.4	42.2	45.3	42.1	41.7	42.8	36.9	45.5	45.5	46.5	46	42.8	41.7	43.3	45.5	47.1	48.1
47.*Om*IFNc3	46.5	42.8	41.7	43.9	47.1	44.9	43.3	43.9	50.3	42.6	50.8	46.5	46	42.2	45.3	43.1	42.7	43.3	39.4	47.1	47.1	46	48.1	43.9	40.6	42.8	46	47.6	47.6
48.*Om*IFNc4	45.9	44.9	44.4	44.9	50.5	48	43.4	45.9	50	44.4	50	46.4	42.9	43.9	43.4	46	44.2	46.9	41.1	43.9	43.9	45.9	44.9	43.9	43.4	44.4	46.4	48.5	47.4
49.*Ss*IFNc1	45.5	42.2	43.3	44.9	49.7	43.9	43.9	43.9	49.7	44.7	50.3	46.5	44.4	42.2	45.3	42.1	41.7	41.7	38.1	47.1	47.1	47.1	47.6	44.4	40.1	41.2	46	47.1	47.1
50.*Ss*IFNc2	43.9	42.8	43.9	43.3	50.3	44.4	42.8	43.3	49.2	43.7	47.1	46	43.9	42.2	45.3	41.6	41.3	41.7	39	46	46	46	46.5	43.3	41.2	42.3	44.9	46	46.5
51.*Ss*IFNc3	44.4	41.7	42.8	44.4	48.7	44.4	42.8	43.3	49.2	44.2	47.1	46	43.9	42.2	45.3	42.1	41.7	41.7	39	46	46	46	46.5	43.3	41.2	41.8	44.9	46	46.5
52.*Dr*IFN2	42.2	46.5	44.6	49.7	47.8	42	46	45.7	47.5	43.2	47.5	44.2	42	44.8	45.3	42.6	43.2	42.5	37.7	46.4	46.4	48.1	45.9	42.1	41.4	44.8	48.1	45.9	47
53.*Dr*IFN3	45.4	43.2	44.1	47	47.8	47	46.5	45.7	44.8	41.6	44.8	51.9	49.7	43.1	47.4	42.6	41.7	45.7	41.1	43.6	43.6	46.4	44.3	46.4	40.9	44.3	44.8	48.1	49.2
54.*Sm*IFN1	42.2	47.6	44.6	44.2	48.4	40.9	48.1	40.3	44.2	46.3	45.9	42.5	45.9	41.4	40.5	37.6	35.9	39.8	35.2	40.3	40.3	38.8	39.5	39.9	36.5	39.2	42	43.1	43.6
55.*Om*IFNf1	40.5	39.5	39.8	41	43.5	40.4	41.2	43	42.2	40.5	40.7	44.9	43.8	42.9	44.2	39.1	39.3	44.6	37.3	42.2	42.2	44.8	41.1	42.6	40.1	43.3	42.5	40.1	44.6
56.*Om*IFNf2	40.5	43.2	42.5	41.3	42.9	40.2	43.9	40.9	45.1	42.1	42.4	42.4	40.2	41.8	42.1	40.1	38.3	45.2	34.7	41.3	41.3	44	42.2	40.2	40.8	45.4	42.4	41.8	42.9

	IFNe							IFNb								IFNc										IFNf	
	30	31	32	33	34	35	36	37	38	39	40	41	42	43	44	45	46	47	48	49	50	51	52	53	54	55	56
1.*Lc*IFNd	23.7	23.2	22.7	24.6	25.1	25.1	25.1	28.6	28.6	28.6	28.6	28.6	27.2	27.2	27.2	24	25.4	25.3	23.8	24	23.5	24	23.1	21.9	20.6	17.7	17.3
2.*Dl*IFN1	24.7	24.1	24.6	26.1	23.9	23.9	25.5	29	28.5	29	29	29	29	29	29	24.6	24.6	24.6	23.9	24.1	25	24.6	23.9	22.7	18.5	21.2	22.7
3.*Sa*IFN1	26.3	25.6	26.1	28.1	25.4	25.4	27	26.8	26.3	26.8	26.8	26.8	25.9	25.9	25.9	24	24	23.5	23.3	23.5	24.4	24	22.7	20.9	19.4	21.1	21.8
4.*Ec*IFN1	24.2	23.6	24.1	24.6	21.9	21.9	21.9	24.9	24.3	24.9	24.7	24.7	24.9	24.9	24.9	22.6	22.6	22.6	23.4	22.1	22.1	22.1	21.2	21.1	22.6	19.8	20.2
5.*Po*IFN1	27.8	27.1	26.1	26.1	25.9	25.9	24.9	27.6	27.6	27.6	27.6	27.6	27.6	27.1	27.1	26	26	27	27	25.6	25.5	26.5	23.5	20.8	21	23	21.2
6.*Ga*IFN1	23.3	21.3	21.3	23.3	23.3	23.3	23.3	24.9	24.9	24.9	24.3	24.3	23.8	24.3	24.3	21.7	23.1	21.2	23.6	22.7	22.2	22.2	19.7	21.1	19.5	21.9	22
7.*On*IFN3	26	26	25	25.5	25.6	25.6	26.2	28.2	27.7	28.7	27.8	27.8	27.5	27.5	27.5	28.6	28.1	28.4	26.7	28.6	27.6	27.6	24	23.4	19.2	20.2	21.9
8.*Ol*IFN	30.5	30.4	29.9	30.9	23.2	23.2	23.2	26.2	25.1	26.2	26.2	26.2	26.2	25.1	25.1	23.1	23.1	22.6	23	23.2	23.7	23.7	23.9	23	17.6	20.7	20.6
9.*Tr*IFN1	25.1	24.3	24.3	24.3	25.1	25.1	24.1	28.6	28	28.6	28.6	28.6	29.1	28	28	25.9	23.8	25.9	23.8	25.4	25.9	25.9	21.2	19.6	22.7	19.3	20.4
10.*On*IFN1	25.8	24.3	23.3	24.8	25.9	25.8	24.7	24.5	24	24.5	24.5	24.5	25.5	24.5	24.5	24	22	22.9	24.4	24.3	23.2	23.8	24.1	21.8	19.5	16.7	17.8
11.*Tn*IFN	25.1	24.2	23.7	23.7	24.2	23.4	23.7	28.6	28.6	28.6	28.6	28.6	28.6	28.6	28.6	26.2	26.2	26.6	25.9	25.6	25.1	25.1	24.2	22.6	22.5	20.8	20.9
12.*Om*IFNd1	32.5	30.7	31.2	31.7	32.6	33.2	32.6	32.1	31.6	32.6	32.6	32.6	32.6	32.1	31.6	27.6	27.4	27.4	27.3	26.4	27.9	27.4	21.2	26.5	20.6	22.8	21.9
13.*Ss*IFNd	31.4	29.8	30.2	30.7	32.1	31.6	31.6	33	32.8	33.5	32.5	32.5	32.5	33	32.5	27	26.9	28.1	25.9	25.9	27.4	26.9	21.6	24.9	22.1	23.2	20.9
14.*Dr*IFN4	22.9	22.1	22.1	21.4	22.9	24.7	23.4	25.3	25.3	25.8	25.3	25.3	24.2	24.7	24.2	22.3	20.8	21.9	22.3	20.8	21.9	21.4	20.1	22.6	19.9	19.3	19.5
15.*Lc*IFNh	25.1	24.8	24.8	24.3	25	25.5	25.1	23.4	22.9	23.9	23.4	23.4	23.9	23.8	23.3	25.2	25.2	24.8	24.5	23.3	22.8	23.3	21.4	23.1	17.2	20.7	22.8
16.*Hb*IFNal3	22.4	22.6	22.6	20.5	21.4	21.4	22.2	23.3	23.7	23.7	23.3	23.3	24.2	23.7	23.3	21.3	20.8	21.8	22	19	19.9	19.4	23.4	20.5	18.3	20.2	19.7
17.*On*IFNal3	23	22.2	21.3	19.6	21.3	21	21.8	23.7	24.2	24.2	23.7	23.7	23.7	23.3	22.8	20.5	20	20.9	21.4	18.2	19.1	18.6	22.8	19.6	18.5	20.6	19.2
18.*Mz*IFNal3	21.1	20.8	20.8	18.1	21.2	21.3	21.8	21.6	22.1	22.1	21.6	21.6	22.5	22.1	21.6	21.5	21.9	23.3	22.7	19.6	20.1	20.1	22.2	20.7	18	22.4	20.9
19.*Pn*IFNal3	21.7	18.7	18.7	16.7	21.3	21.1	21.1	24.4	24.8	24.8	24.4	24.4	23	23	22.6	18.3	19.3	20.6	21.8	18.5	18.9	18.9	19.3	21.7	16.6	21.1	16.2
20.*Ca*IFN	30	27.7	28.2	27.7	26.8	26.8	26.8	25.8	25.8	25.8	25.8	25.8	25.3	25.8	25.8	25.9	25.9	25.4	24.3	25.9	25.9	25.9	20	20.4	17.1	21.5	18.8
21.*Ci*IFN	30	27.7	28.2	27.7	26.8	26.8	26.8	25.8	25.8	25.8	25.8	25.8	25.3	25.8	25.8	25.9	25.9	25.4	24.3	25.9	25.9	25.9	20	20.4	17.1	21.5	18.8
22.*Cc*IFN	28.1	26.5	27	26.1	29	29	28	25.4	25.9	25.4	25.4	25.4	25.9	25.4	24.9	27.9	26.3	25.6	25.6	25.8	26.3	26.3	21.8	22.6	19.1	25.7	23.3
23.*Mp*IFN	28.9	26.7	27.2	27.2	26.8	26.8	26.8	24.9	24.9	24.9	24.9	24.9	24.4	24.9	24.9	25.3	25.3	24.7	24.2	25.3	25.3	25.3	19.6	20.7	16.8	20.2	19.2
24.*Cm*IFN	28.4	26.2	26.7	27.7	27.9	28.4	27.9	27.7	27.2	27.7	28.3	28.3	27.7	27.7	27.7	22.8	22.3	23.8	22.3	22.6	22.6	22.6	21.1	22.9	19.7	23	20.2
25.*Ip*IFN	24.7	23	21.5	22.5	25.1	24.6	25.9	21.3	21.8	21.3	21.8	21.8	21.3	21.3	21.3	21.1	20.1	22.2	21.8	20.1	19.6	20.1	20.1	23	18.2	18.6	19.7
26.*Ip*IFN2	28.7	26.8	27.2	28.6	28.4	28.9	28.4	22.8	23	22.8	23.4	23.4	24.6	22.8	22.8	24	24.1	24.6	24.5	24.1	24	23.6	24.3	25.4	22.7	20	20.7
27.*Dr*IFN1	27.2	26.1	26.1	25.2	28.4	28.4	28.4	23.8	22.8	23.8	23.8	23.8	23.3	23.3	23.3	25.5	25.4	23.8	25.9	23.5	23.5	23.5	22.7	23.2	19.2	20.6	20.7
28.*Om*IFNa1	27.6	25.5	26.8	27.5	27	27.5	26.5	30.3	29.3	30.3	30.9	30.9	30.9	29.8	29.8	26.4	26.9	27.6	27.8	26.6	25.9	25.4	26.2	26	19.3	23	22.5
29.*Ss*IFNa1	30	27.7	29.1	29.7	29.1	29.1	28.6	31.4	30.3	31.4	31.9	31.9	31.4	31.4	30.9	27	28.4	27	29.6	26	26	25.5	26	27.1	21.2	24	23.5
30.*Om*IFNe1		92.9	91.9	86.9	69.5	68.4	67.4	43.5	44.1	44.1	43	43	44.6	44.6	44.6	41.7	42.8	43.9	42.3	43.3	44.4	44.4	45.2	43	39.8	19.1	19.9
31.*Om*IFNe2	93.4		96.5	91.4	64.3	63.3	62.3	42.4	42.9	42.9	41.9	41.9	43.4	43.4	43.4	40.4	40.4	40.9	43.4	40.4	41.4	41.4	43.9	39.9	38.4	18.1	19.2
32.*Om*IFNe3	93.4	98.5		91.9	65.8	64.8	63.8	42.9	43.4	43.4	42.4	42.4	43.9	43.9	43.9	41.9	41.4	41.4	42.4	42.4	41.9	41.9	43.4	40.4	37.4	18.4	19.3
33.*Om*IFNe4	92.2	94.9	95.5		65.3	64.3	63.3	45.1	45.6	45.6	45.1	45.1	45.1	45.1	45.1	40.4	40.4	40.9	41.3	40.9	41.5	41.5	40.4	41.5	38.3	17.3	18.5
34.*Om*IFNe5	84.4	78.8	78.8	79.3		97.8	97.3	48.4	48.9	48.4	48.4	48.4	48.4	48.4	48.4	46	46.5	45.5	43.9	46	46	46	44.1	43.5	44.6	18.5	20.2
35.*Om*IFNe6	84.4	78.8	78.8	79.3	98.9		96.2	48.9	49.5	48.9	48.9	48.9	47.8	47.8	47.8	45.5	46	44.4	42.9	45.5	46	46	44.1	43.5	43	18.5	19
36.*Om*IFNe7	84.4	78.8	78.8	79.3	98.9	97.8		47.8	48.4	47.8	48.9	48.9	48.4	47.3	47.3	45.5	46	44.4	43.9	45.5	45.5	45.5	43	45.2	45.2	19	20.2
37.*Om*IFNb1	21.3	21.1	21.6	22	21.4	21.9	20.9		97.8	98.9	98.9	98.9	95.1	96.7	96.2	35.2	33.7	34.2	31.7	33.7	33.7	33.7	32.1	31.4	24	24.9	27.1
38.*Om*IFNb2	20.8	20.6	21.1	21.5	21.4	21.9	20.9	98.4		96.7	96.7	96.7	94.5	96.2	95.6	35.7	34.2	34.7	32.2	34.2	34.2	34.2	32.1	31.9	23.5	24.9	26.6
39.*Om*IFNb3	21.8	21.6	22.1	22.5	22.4	23	21.9	100	98.4		97.8	97.8	95.1	96.7	96.2	34.4	32.8	33.3	30.9	32.8	32.8	32.8	32.1	30.9	24	24.9	26.6
40.*Om*IFNb4	20.8	20.6	21.1	22	21.4	21.9	20.9	99.5	97.8	99.5		100	94	95.6	95.1	35.7	33.7	34.2	31.7	33.7	33.7	33.7	31.6	30.9	23.7	25.4	27.7
41.*Om*IFNb5	20.8	20.6	21.1	22	21.4	21.9	20.9	99.5	97.8	99.5	100		94	95.6	95.1	35.7	33.7	34.2	31.7	33.7	33.7	33.7	31.6	30.9	23.7	25.4	27.7
42.*Ss*IFNb1	20.8	20.6	21.1	22.5	22.4	23	21.3	96.7	96.7	96.7	96.2	96.2		97.3	96.7	34.7	33.2	33.7	31.2	33.2	33.2	33.2	32.1	31.9	24	23.4	26.2
43.*Ss*IFNb2	20.4	20.2	20.7	22	21.9	22.4	20.8	97.3	97.3	97.3	96.7	96.7	98.4		99.5	34	32.7	33.2	30.7	33.2	32.7	32.7	32.6	31.4	24	25.1	26.7
44.*Ss*IFNb3	20.4	20.2	20.7	22	21.9	22.4	20.8	97.3	97.3	97.3	96.7	96.7	98.4	100		33.5	32.1	32.7	30.2	32.7	32.1	32.1	32.1	30.9	24	24.6	26.2
45.*Om*IFNc1	21.9	22.6	23	24	20.9	20.9	20.5	56.7	57.2	56.1	57.2	57.2	55.6	54.5	54.5		96.3	94.1	84.2	90.4	91.4	92	38.3	44.1	34.4	22.8	23.6
46.*Om*IFNc2	22.5	22.6	23	24	20.3	20.3	19.9	55.1	55.6	54.5	55.6	55.6	54.5	54	54	96.8		95.7	85.7	92	93	93.6	37.3	43.6	34.7	22.7	22.2
47.*Om*IFNc3	23.5	21.9	23.6	24.5	20.8	21.8	20.4	55.6	56.1	55.1	56.1	56.1	55.1	54.5	54.5	96.3	97.3		86.7	92	92.5	93	38.9	44.1	34.2	23.7	23.2
48.*Om*IFNc4	23.1	22.3	21.3	22.3	19.2	19.2	20.7	51.5	52	51	52	52	51	50.5	50.5	88.8	89.8	90.8		80.6	81.1	81.6	35	42.6	34	21.7	22.4
49.*Ss*IFNc1	23	21.4	22.2	25	20.8	20.8	20.4	55.1	55.6	54.5	55.6	55.6	54.5	54	54	94.7	96.3	96.8	88.3		96.3	96.8	38.7	44.1	33.7	20.9	20.5
50.*Ss*IFNc2	23	21.4	22.6	25	20.8	20.8	20.4	54.5	55.1	54	55.1	55.1	54	53.5	53.5	96.3	97.9	97.9	89.3	97.9		99.5	37.3	43.1	32.7	20.9	21.7
51.*Ss*IFNc3	23	21.4	22.6	25	20.8	20.8	20.4	54.5	55.1	54	55.1	55.1	54	53.5	53.5	96.3	97.9	97.9	89.3	97.9	100		37.8	43.1	32.7	20.9	21.7
52.*Dr*IFN2	25.1	25.2	25.2	22.1	21.9	21.9	21.9	51.4	51.9	51.4	51.4	51.4	51.9	51.9	51.9	61	62	62.6	57.7	62	61	61		41.1	24.5	21.2	22.6
53.*Dr*IFN3	20.8	21.7	21.7	22.3	21.1	21.1	21.8	51.9	52.5	51.9	51.9	51.9	52.5	52.5	52.5	64.2	64.2	63.6	59.2	62	61.5	62	65.2		26.8	22.8	24.1
54.*Sm*IFN1	19.1	18	18.4	18.4	19.4	18.9	19.4	48.1	47.5	48.6	47.5	47.5	48.1	48.6	48.6	57.2	57.8	58.3	56.1	58.3	58.3	58.3	49.7	50.3		20.6	20.8
55.*Om*IFNf1	40.3	39.4	38.4	38.9	43	41.9	43	43.7	43.2	43.7	44.3	44.3	43.7	43.7	43.7	43.3	43.3	43.9	44.4	43.3	42.2	42.2	48.6	50.3	43.1		75
56.*Om*IFNf2	43	40.9	41.9	43	45.2	43.5	44.6	42.4	41.8	42.4	42.9	42.9	42.9	42.9	42.9	43.9	43.3	44.4	45.4	44.9	42.8	42.8	48.9	49.5	45.1	81	

Supplementary Table 2 | Comparison of identities (top right) and similarities (bottom left) of large yellow croaker IFNd and IFNh with other teleost type I IFNs. The sequence identities/similarities of IFNds between Perciforme species are highlighted in yellow and bold. *Lc, Larimichthys crocea; Dl, Dicentrarchus labrax; Sa, Sparus aurata; Ec, Epinephelus coioides; Po, Paralichthys olivaceus; Ga, Gasterosteus aculeatus; On, Oreochromis niloticus; Ol, Oryzias latipes; Tr, Takifugu rubripes; Tn, Tetraodon nigroviridis; Om, Oncorhynchus mykiss; Ss, Salmo salar; Hb, Haplochromis burtoni; Mz, Maylandia zebra; Pn, Pundamilia nyererei; Ca, Carassius auratus; Ci, Ctenopharyngodon idella; Cc, Cyprinus carpio; Mp, Mylopharyngodon piceus; Cm, Cirrhinus molitorella; Ip, Ictalurus punctatus; Dr, Danio rerio*.
