# Supplementary material for: Identification of Two Subgroups of Type I IFNs in Perciforme Fish Large Yellow Croaker Larimichthys crocea Provides Novel Insights into Function and Regulation of Fish Type I IFNs
Source: Front Immunol. 2016 Sep 7;7:343. doi: 10.3389/fimmu.2016.00343 (PMC5013148; doi:10.3389/fimmu.2016.00343)
Supplement: Supplementary file 3 [file image_1.pdf]

Supplementary Figure 1

Identification of Two Subgroups of Type I IFNs in Perciforme Fish Large Yellow Croaker *Larimichthys crocea* Provides Novel Insights into Function and Regulation of Fish Type I IFNs

Yang Ding*, Jingqun Ao, Xiaohong Huang

*** Correspondence:** Xinhua Chen: chenxinhua@tio.org.cn

Supplementary Figure 1A

1 GTGAGGAAAATGAAATAGGTGCGTCCTGCTACAGTATAAATGAGCCGCTGCAGGTGAGTT
61 TGAACACAACACCTGGACACATCCGACTTTCGCCACTCAAAGACACTTTGTCTGTTTGTA
121 AAG**ATG**CTCAGCAGGATCTTGTTTGTGTGCCTGTCTCTCAGTCTGTACAGTGCAGGCTCC
1 M L S R I L F V C L S L S L Y S A G S
181 TCGCTAAGCTGCAGATGGGTGGATCATAAATTCAGACAGCACAGTGAAACTTCTCTGGAT
20 S L S C R W V D H K F R Q H S E T S L D
C1
241 CTACTCAACACCATGGCTAATAACTCCACTAACAGCACTGAGGATGCTCAGGTGGAGGAC
40 L L N T M A N N S T N S T E D A Q V E D
301 ACTGTGGCCTTCCCTAATGATCTGTACAGCCAGGCGTCCAAAGCATCAGCTGAGGATAAA
60 T V A F P N D L Y S Q A S K A S A E D K
361 CTTCATTTCACAGTTCAGGTTCTGGAGGAGGCGGCTGCCCTGTTTGAGGAGGATCACAGC
80 L H F T V Q V L E E A A A L F E E D H S
421 AATGCTTCATGGGAGGAGAACACAGTGGAGAACTTTGTCAATGTTGTAAACCAGCAGGCT
100 N A S W E E N T V E N F V N V V N Q Q A
481 GACGGCCTTCGCTCCTGTACTGGGAGTCACGGCCACAAGAAGAAGAACAAGAAGCTGCAC
120 D G L R S C T G S H G H K K K N K K L H
C3
541 ATGTATTTCAAGAGACTGTCGAGTCACGTCCTGAAGAAGATGAGCCACAGTGCTGAAGCC
140 M Y F K R L S S H V L K K M S H S A E A
601 TGGGAGCTGATCAGGAAGGAGATCAGGACCCATCTGATGAGAGCAGACCAGCTGGTTTCA
160 W E L I R K E I R T H L M R A D Q L V S
661 TCTCTGCGCAACACCAAC**TAA**AATCTGTAATCTGACTGTCAGATGAACAATCTGTTTATT
180 S L R N T N *
721 TATTGACATGAAGAAT**ATTTA**TTAACTT**ATTTA**TTTATTGAATCATTGAGCAGTTTGTGT
781 **ATTTA**TTAGTTGATTGATTGAATATTCTACAAAACTACTATTTTTATAATGAATATATTG
841 TTGATCAACTT**ATTTATTTATTTA**AGTTATTTTTTTTACATGGATACCTGCATAAAAC*AA*
901 *TAAA*AATGAGACTTTCTCCAGCAAAAAAAAAAAA

Supplementary Figure 1B

1	TCATCTTCGTCATCTTCGTCATCTTCATC**ATG**GTTAACTGGACCGGCGTGCTCTTCGTCC
1	M V N W T G V L F V
61	TCTGTGGGACCCTCCTGACTCCTGCACTCTGCTGTGATTGGCTCAGACACTACGGTCACC
11	L C G T L L T P A L C C D W L R H Y G H
	C1
121	TGAGCAACGACTCATTGACTCTCCTCCAGCAGATGGGCGGTCAGTGGACTGGACAGGAGT
31	L S N D S L T L L Q Q M G G Q W T G Q E
181	GTCGAGTTCCCTTTCCAAGAAAAATCTACAGAGACATATATAAGGCTGAGGTGCAGTCCC
51	C R V P F P R K I Y R D I Y K A E V Q S
241	AGTTGGTTTTCATCAGAGACAGTCTGAAGCTGATTTCTGGTCTCTATCACCATGACAACC
71	Q L V F I R D S L K L I S G L Y H H D N
301	TCACCTCTGCTTCCTGGGACACCGTCAAGACCGAACACTTCCTGATAAGCATCCACAGAC
91	L T S A S W D T V K T E H F L I S I H R
361	AGACAGAAGAACTCAACACCTGTGTGTTGGCGAACAAGACGTCCAACAGCAGTCTGAGAA
101	Q T E E L N T C V L A N K T S N S S L R
	C3
421	AGTACTACAGGAGACTGGCCAGGAGTACTCTGCACTGCACTGGTGGCAGTCCTGCGTCGT
121	K Y Y R R L A R S T L H C T G G S P A S
481	GGGAGCTGATCAGGAAACAGACCAAACTGCACCTGGATCAGTTGGACCTGCTGGTGGAGT
141	W E L I R K Q T K L H L D Q L D L L V E
541	GCATCAAGAGTTCATCTGCCGCCTGCAGGAGGCGCTCTGCAGCGAGTGGACGGCAGCAC**T**
161	C I K S S S A A C R R R S A A S G R Q H
601	**GA**CTGTTCAGAGTGTTTCACTGATTCTGTCTCTCTGTGAGAGTTTGTCTTCTATTTCACT
181	*
661	CAGGTTTTTT**ATTTA**TTGATGGACACACCGCTCGTCAT**ATTTA**TTATTATTAT**ATTTATT**
721	**TATTTA**ATTTTTTTATAAACTGTACTTTATACTGTACTGTATATTTGTGATAACTGACAA
781	*AATAAA*GATGCTTCACGCTCAAAAAAAAAAAAA

Supplementary Figure 1C

```
                          NF-κB
-877    CTGAGTCCTCGGAGTTTCCCGAGAAAACCGAAAACCCCTCCGTGAGCGTGTCAGACAGCT
```

```
-997    ACAAACGTCATAAATATAAGTGTGTGTGTGAAGTGTGCGCATGCGCATGCGCGTGTTTGA
-937    CCCCACCTAGCGGGCCCGGGCTGGGATGAAACGTGATGACGTCACATTTGAAAAACGAAA
                          NF-κB
-877    CTGAGTCCTCGGAGTTTCCCGAGAAAACCGAAAACCCCTCCGTGAGCGTGTCAGACAGCT
-817    CTGTGACAGGAAGCCACCGTGGTGGTATTTAAAAGTATGAGCAGCTGAGATTGAATGACA
-757    CTTTGTCACGAAGCTTCAGGAGGAACAAACGAGCCATTATCTCCGACTTTCTGGCCCTGC
-697    ACCTGAACTCAACACGCACCTACAGCGACTGGAAGCTCGTGACTGCAGGTAGGAGAGTTT
-637    GCAATGCTGTGAAATATGATTAATAATGTATTCATTTATAGCACATCTGCAGACTGAGTA
-577    ACGAAATATACCTTGTGTACTATGTATGAAATTTCACACTTGAGTTGTTGTACTCGAGTA
                                              ISRE
-517    TTTCCACATTACGTAACTTTATAGCCTGTTTCACTTCTATTTTAAGTTACAATGAAAAAA
-457    CAAATTGATCTGTTATTGTACACTAATATTCATATCTGACACTTGAATGAATAAAATGCA
-397    TGAATATGTATTTTTACCTTTCATGCTTAAATACATGAACTGTAACAGTTACAGTGTTTT
                  AP1
-337    TAAGTTTTGTTTTACTCATATTTAGTATTTTTGGAATTGAAGGCTGAACCAAACCTGGCA
-377    TCATATTTGCCTGCTTCCTCTTTTTGGTTATTCAGAAATTGTAAATATAAGTTAACTTCC
-317    ACCAGCTGCATCATAGCAATGCACACATCAGGTTGTTGATGCTCAGATGTGTGTACCTAT
-257    TCCTGATTCCTGTGTAAGTGAGTCCTTGCCTTGACATATTTTGCCAATAAATCTGATTTA
-197    GTAGTAGTGAAAAGTGTCATCCCTCACTTGCATGAGGATCTTTTAAAAATAGATTTTCTC
                                                   ISRE
-137    ATTTTAAATTAATATCAAACATGCAAAAGTTAAGTTACTTTTTTTCTACTAATTCCAGGCA
-77     GGACGTGATGATTACATTTCTTTCTGCATGTATCTGGTTGTAAAGTGACTAAATAGTGCC
                          +1
-17     ATCTACAGGGCCTGAACATTCTAATCATTAATATAAACCTATAAATACATATGAATAATT
                                                               AP1
44      GTGGTGATTAAACATTCAGCCTGGCTCAGCTTGTTTTATTTACATAAAGTGAATCATCAA
104     CAATTATTTCTGTTTCACTGTTGGATTCAGGGCAAACTGCTCTGGATACTGAGAGCTACA
164     AGACAAGAAATATATATATATTTTTAATCATAGATGATCTGACACTGTTGATGTGACTTT
224     TTTTTTTTTGCTCCTGCAGGTATG
```

Supplementary Figure 1 | Sequences analysis. (A, B) Nucleotide and deduced amino acid sequences of large yellow croaker IFNd (A) and IFNh (B). The stop codon is represented with an asterisk. The motifs associated with mRNA instability (ATTTA) are shown in bold and underlined. The typical mRNA polyadenylation signal (AATAAA) is shown in italic and underlined. In the deduced amino acid sequence, the signal peptide is underlined. The amino acid residues across the introns in the coding region are indicated by tringles. The signature motifs of type I IFNs are shaded in grey. The cysteine residues are boxed and the two conserved cysteine residues (C1 and C3) of group I type IFNs are indicated by extra arrows. **(C)** Predicted transcription factor binding sites of large yellow croaker IRF3 promoter. Large yellow croaker IRF3 gene and promoter were obtained from Genbank (**KX148469**). The 1 kb 5'-flanking sequence of the predicted transcription start site was analysed by the MatInspector program. The important transcription factor binding sites such as ISRE, NF-κB, and AP1 were highlighted and underlined. The analysis revealed that two predicted ISRE motifs are present in the promoter of large yellow croaker IRF3.
