# Supplementary material for: Identification of Two Subgroups of Type I IFNs in Perciforme Fish Large Yellow Croaker Larimichthys crocea Provides Novel Insights into Function and Regulation of Fish Type I IFNs
Source: Front Immunol. 2016 Sep 7;7:343. doi: 10.3389/fimmu.2016.00343 (PMC5013148; doi:10.3389/fimmu.2016.00343)
Supplement: Supplementary file 4 [file image_2.pdf]

Supplementary Figure 2

Identification of Two Subgroups of Type I IFNs in Perciforme Fish Large Yellow Croaker *Larimichthys crocea* Provides Novel Insights into Function and Regulation of Fish Type I IFNs

Yang Ding*, Jingqun Ao, Xiaohong Huang

*** Correspondence:** Xinhua Chen: chenxinhua@tio.org.cn

Supplementary Figure 2

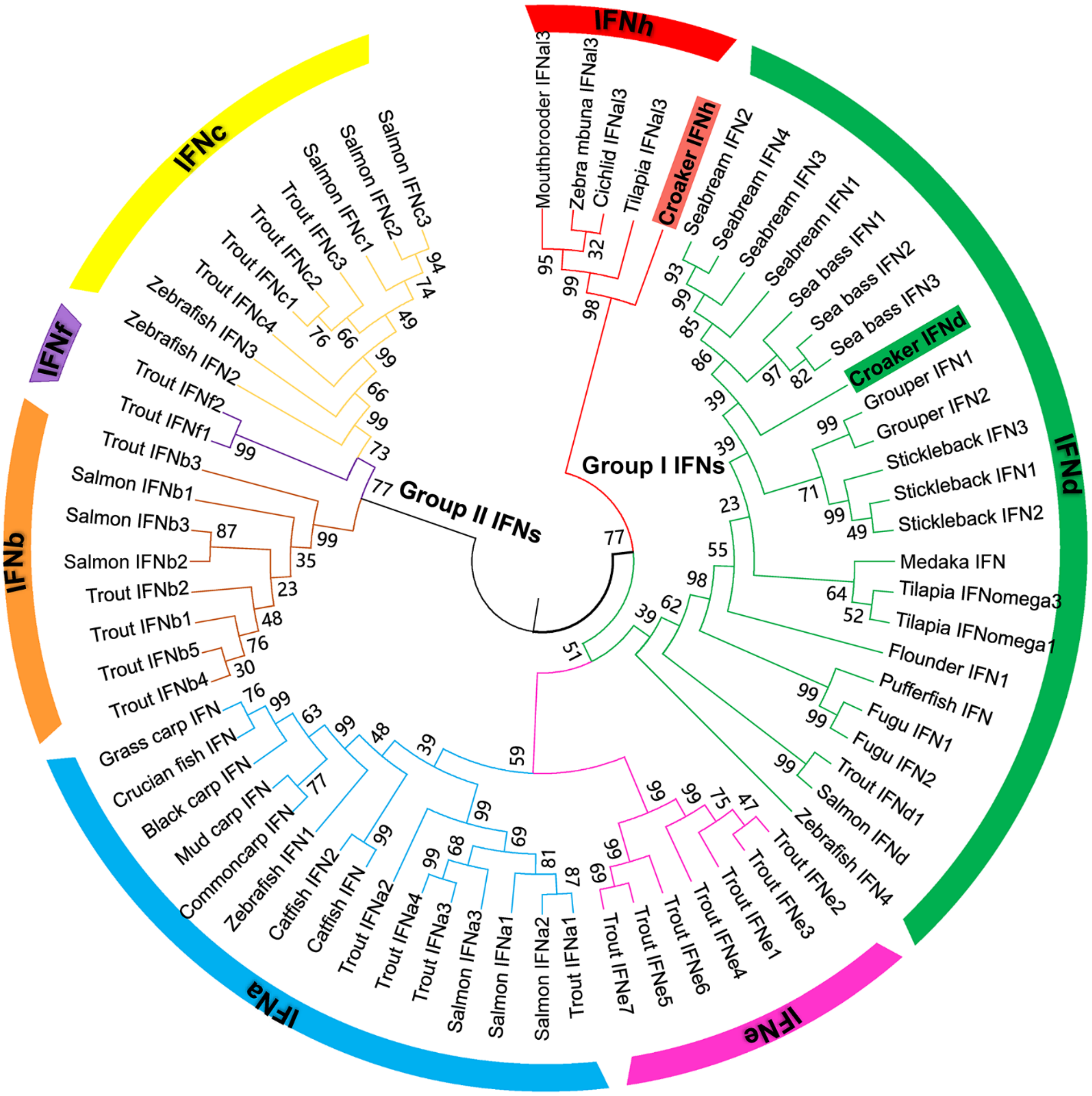

Supplementary Figure 2 | Phylogenetic tree of fish type I IFN family members based on the genetic distances of deduced amino acid sequences. Deduced amino acid sequences of type I IFN family members were aligned and the tree was constructed with the Minimum Evolution method using the MEGA (version 6) software package. The tree is bootstrapped 10,000 times, and the bootstrap values of the major branches are shown as percentages.
