# Supplementary material for: Identification of Two Subgroups of Type I IFNs in Perciforme Fish Large Yellow Croaker Larimichthys crocea Provides Novel Insights into Function and Regulation of Fish Type I IFNs
Source: Front Immunol. 2016 Sep 7;7:343. doi: 10.3389/fimmu.2016.00343 (PMC5013148; doi:10.3389/fimmu.2016.00343)
Supplement: Supplementary file 5 [file image_3.pdf]

Supplementary Figure 3

Identification of Two Subgroups of Type I IFNs in Perciforme Fish Large Yellow Croaker *Larimichthys crocea* Provides Novel Insights into Function and Regulation of Fish Type I IFNs

Yang Ding*, Jingqun Ao, Xiaohong Huang

*** Correspondence:** Xinhua Chen: chenxinhua@tio.org.cn

Supplementary Figure 3

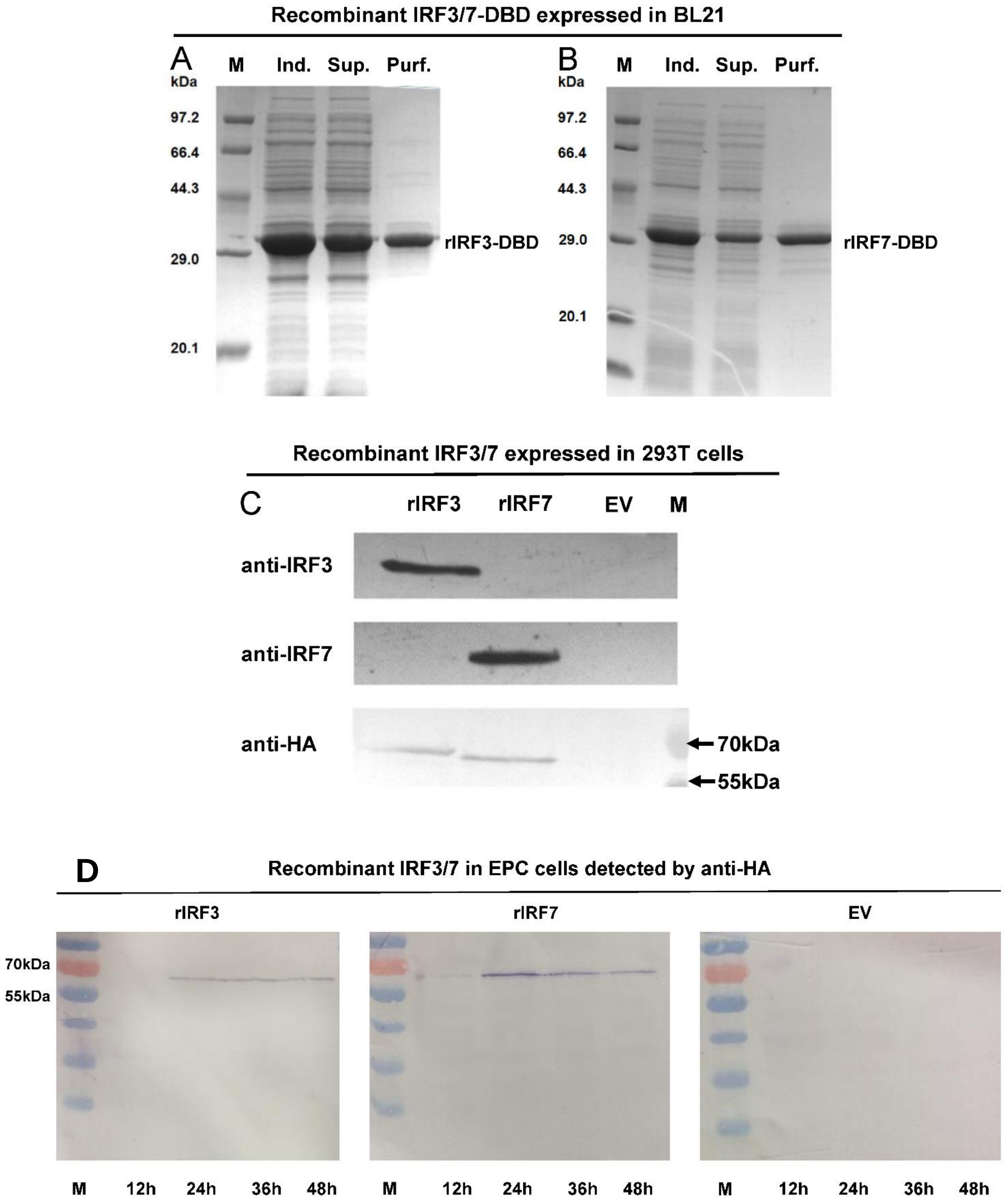

Supplementary Figure 3 | Specificity of polyclonal anti-IRF3 and -IRF7 antibodies. (A, B) Production and purification of recombinant DBD of large yellow croaker IRF3 and IRF7 (rIRF3-DBD and rIRF7-DBD). The rIRF3-DBD (A) and rIRF7-DBD (B) were expressed as fusion protein with thioredoxin (Trx) and hexahistidine (6×His) tag in *E. coli* BL21. **M**: Standard protein molecular weight marker. **Ind.:** The lysates of induced bacteria. **Sup.**: Supernatants of lysates. **Purf.**: The purified proteins. The purified proteins were injected into white New Zealand rabbit to raise polyclonal Abs against large yellow croaker IRF3 and IRF7. **(C)** Specificity of polyclonal anti-IRF3 and anti-IRF7 antibodies. HEK293T cells were transfected with pCMV-HA-IRF3, pCMV-HA-IRF7, or pCMV-HA (as control) for 48 h. The lysates of these transfected cells were analysed by Western-blotting using the indicated polyclonal Abs, respectively. **(D)** Recombinant large yellow croaker IRF3 and IRF7 in EPC cells. EPC cells seeded in 6-well plates were transfected with 3 μg of pCMV-HA-IRF3, pCMV-HA-IRF7, or pCMV-HA, respectively. At 12, 24, 36, and 48 h post-transfection, the supernatants of the transfected cell lysates were analysed by Western-blotting using the anti-HA antibody. **M**: Standard protein molecular weight marker.
